# A Bivalent Activatable Fluorescent Probe for Screening and Intravital Imaging of Chemotherapy‐Induced Cancer Cell Death

**DOI:** 10.1002/ange.202113020

**Published:** 2021-12-16

**Authors:** Nicole D. Barth, Lorena Mendive‐Tapia, Ramon Subiros‐Funosas, Ouldouz Ghashghaei, Rodolfo Lavilla, Laura Maiorino, Xue‐Yan He, Ian Dransfield, Mikala Egeblad, Marc Vendrell

**Affiliations:** ^1^ Centre for Inflammation Research The University of Edinburgh UK; ^2^ Laboratory of Medicinal Chemistry Faculty of Pharmacy and Institute of Biomedicine (IBUB) University of Barcelona Spain; ^3^ Cold Spring Harbor Laboratory Cold Spring Harbor NY 11724 USA

**Keywords:** apoptosis, breast cancer, chemotherapy, fluorogenic probes, peptides

## Abstract

The detection and quantification of apoptotic cells is a key process in cancer research, particularly during the screening of anticancer therapeutics and in mechanistic studies using preclinical models. Intravital optical imaging enables high‐resolution visualisation of cellular events in live organisms; however, there are few fluorescent probes that can reliably provide functional readouts in situ without interference from tissue autofluorescence. We report the design and optimisation of the fluorogenic probe Apotracker Red for real‐time detection of cancer cell death. The strong fluorogenic behaviour, high selectivity, and excellent stability of Apotracker Red make it a reliable optical reporter for the characterisation of the effects of anticancer drugs in cells in vitro and for direct imaging of chemotherapy‐induced apoptosis in vivo in mouse models of breast cancer.

## Graphical abstract

We describe Apotracker Red as a single fluorescent activatable probe for robust quantification of cancer cell death in fluorescence‐based assays and in vivo using multiphoton intravital imaging in mouse models of breast cancer.

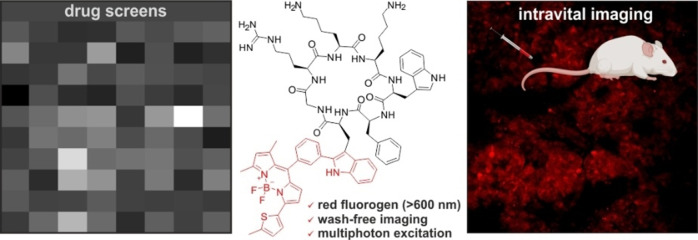

## Introduction

Cancer research is rapidly evolving and new therapeutic approaches—including immunotherapies and combination therapies—are in high demand. In line with this progress, technologies that can reliably determine and quantify the efficacy of anticancer drugs across platforms and in multiple biological assays are urgently needed.^[1]^ Ideally, these technologies should enable rapid screening of bioactive compounds as well as facilitate mechanistic studies in preclinical models to validate drug action in vivo. To this end, the emergence of intravital imaging in mouse models of cancer holds great promise to study how cancer cells interact within the tumour microenvironment in vivo and to analyse how these interactions can be effectively perturbed to improve therapeutic outcome.^[2]^

Anticancer therapeutic strategies aiming at the full eradication of tumour cells are continuously under development, and the extent of cancer cell death is one of the most reliable indicators of drug efficacy.^[3]^ Several technologies have been described to monitor cancer cell death but there are few that can be used across high‐throughput screenings in vitro and validation studies in vivo.^[4]^ Most screening assays rely on well‐established markers of cell death (e.g., nuclear dyes,^[5]^ annexins,^[6]^ caspase‐activated fluorophores^[7]^), which cannot always be used in vivo because of the need for washing steps, limited chemical stability or poor biodistribution.^[8]^ Alternatively, intravital imaging studies of cancer cell proliferation employ transgenic fluorescent proteins^[9]^ that are hard to translate to human studies and are not always compatible with patient‐derived cell lines. The development of new and versatile fluorescent reporters that can track the fate of cancer cells without the need for genetic modification would significantly accelerate the translation of anticancer therapies to in vivo models of cancer.

Fluorescent peptides are versatile structures for imaging of biological events with high spatial and temporal resolution in a broad range of experimental conditions.^[10]^ Peptides and proteins with high affinity for biomolecules present in apoptotic cells but not in viable cells (e.g., cell surface‐exposed phosphatidylserine (PtdSer) headgroups) have been reported in molecular imaging studies to monitor the efficacy of anticancer therapies.^[11]^ Therefore, we envisaged that the chemical optimisation of peptides targeting such biomarkers with activatable fluorogenic reporters^[12]^ could provide new optical tools to quantify the extent of apoptosis induced by anticancer drugs and to perform in vivo intravital imaging experiments.

**Scheme 1:**
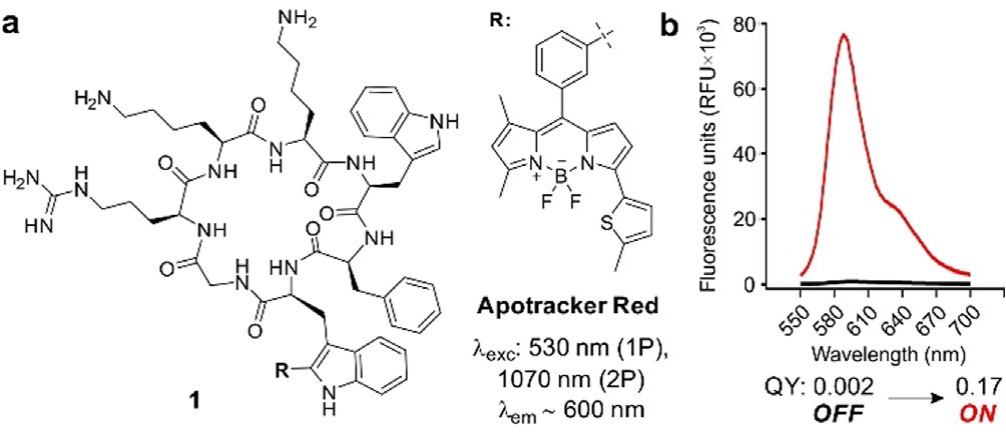
a) Chemical structure and photophysical properties of the fluorogenic probe Apotracker Red (one‐photon excitation (1P), two‐photon excitation (2P)). b) Fluorescence emission of Apotracker Red (40 μM) in phosphate buffered saline (PBS, black) and in suspensions of liposomes (3.75 mg mL^−1^) in PBS (red). Excitation wavelength: 530 nm. Fluorescence quantum yields (QY) were determined as relative to the standard Rhodamine 101.

Our group has previously reported green fluorescent probes for imaging of apoptotic bodies and apoptotic cells by incorporating the Trp‐BODIPY fluorophore (emission maximum ≈520 nm) into different cyclic peptide structures.^[13]^ These probes are useful for confocal microscopy but their short emission wavelengths compromise their utility for multiphoton in vivo imaging, especially in tissues with potential autofluorescence such as tumours.^[14]^ To address this limitation, we designed a new probe termed Apotracker Red in which we derivatised the PtdSer‐binding cyclic peptide 1 with a red fluorogenic amino acid (emission maximum ≈600 nm, Scheme 1) that emits only after binding to apoptotic cancer cells but remains silent (quantum yield (QY)<1 %) in viable cancer cells (full synthetic details in Supporting Information). Notably, the longer wavelengths of Apotracker Red make it compatible with multiphoton excitation (≈1070 nm) for intravital imaging with an emission profile distinguishable from tissue autofluorescence and second‐harmonic generation signals found in tumours.

## Results and Discussion

### Apotracker Red is a fluorogenic peptide that binds to PtdSer on the surface of apoptotic cells

We designed the chemical synthesis of Apotracker Red in two main steps: 1) solid‐phase peptide synthesis (SPPS) of the protected linear sequence^[15]^ including the BODIPY fluorogenic amino acid (Scheme 1), and 2) head‐to‐tail cyclisation followed by deprotection to obtain the final Apotracker Red probe (Supporting Information, Figure S1). Given the lability of the BODIPY scaffold to acidic conditions,^[16]^ we designed an approach where the side chains of Lys and Arg were protected with allyloxycarbonyl (Alloc) groups, which could be removed by reaction with Pd^0^ complexes without affecting the BODIPY core (Figure S2). Notably, this procedure resulted in a significant improvement of the purity of the crude peptides prior to HPLC purification (Figures S2 and S3) and the overall recovery yields (22 % yield for Apotracker Red). The simplicity of this methodology and compatibility with commercially available building blocks (e.g., Alloc‐ and OAll‐protected amino acids) makes it an efficient approach for the scalable SPPS of BODIPY‐labelled fluorescent peptides, overcoming cumbersome purification steps and low yields (i.e., typically <5 %, as reported in other examples).[^10^, ^13b^, ^16a^]

**Figure 1:**
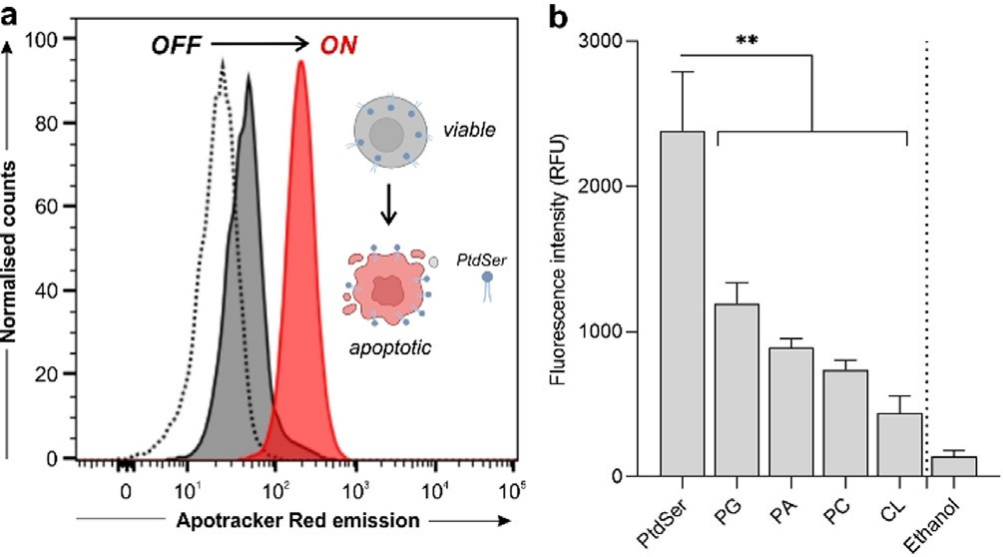
Binding selectivity and spectral properties of Apotracker Red. a) Fluorescence histograms of Jurkat T cell suspensions (unstained cells (dotted), live cells (dark grey), staurosporine‐treated cells (red)) after incubation with Apotracker Red (150 nM, 15 min) under wash‐free conditions. Representative plots from 3 independent experiments. b) Fluorescence intensity of Apotracker Red (excitation wavelength: 530 nm) after incubation with phospholipid‐coated well plates at 1 mg mL^−1^ (phosphatidylglycine (PG), phosphatidic acid (PA), phosphatidylcholine (PC), cardiolipin (CL)) and ethanol as a negative control. Data presented as mean±SEM (standard error of the mean, *n*=5). *P* values obtained from one‐way ANOVA using multiple comparisons (** for *p*<0.01).

Next, we examined the optical properties of Apotracker Red in different microenvironments mimicking those found in apoptotic cancer cells. First, we recorded the fluorescence emission of Apotracker Red in suspensions of viable as well as apoptotic cancer Jurkat cells. As shown in Figure 1 a, Apotracker Red showed bright fluorescence emission in apoptotic Jurkat cells whereas it remained non‐fluorescent in live cells. The plasma membrane of cancer cells undergoes significant remodelling at early stages of cell death, and phospholipids containing PtdSer headgroups are rapidly exposed to the outer leaflet of the cells. PtdSer is a known biomarker of apoptotic cells,^[17]^ and can be targeted by imaging reagents[^6^, ^18^] without requiring cell permeabilisation or fixation. Therefore, we examined the fluorescence profile of Apotracker Red upon incubation with different phospholipids to examine its selectivity for PtdSer (Figure 1 b).

Apotracker Red displayed significantly brighter fluorescence after interaction with PtdSer when compared to other phospholipids (Figure 1 b; Figure S4), including lipids that are exposed on the surface of cell membranes as well as negatively charged lipids that could interact non‐specifically with positive charges in the Apotracker Red structure (i.e., Lys and Arg residues). Altogether, these results confirmed that Apotracker Red displays strong fluorogenic behaviour in the red region of the visible spectrum upon interaction with PtdSer on the surface of apoptotic cancer cells, enabling their detection under wash‐free conditions.

### Apotracker Red rapidly and selectively stains apoptotic cells but not viable cells

**Figure 2:**
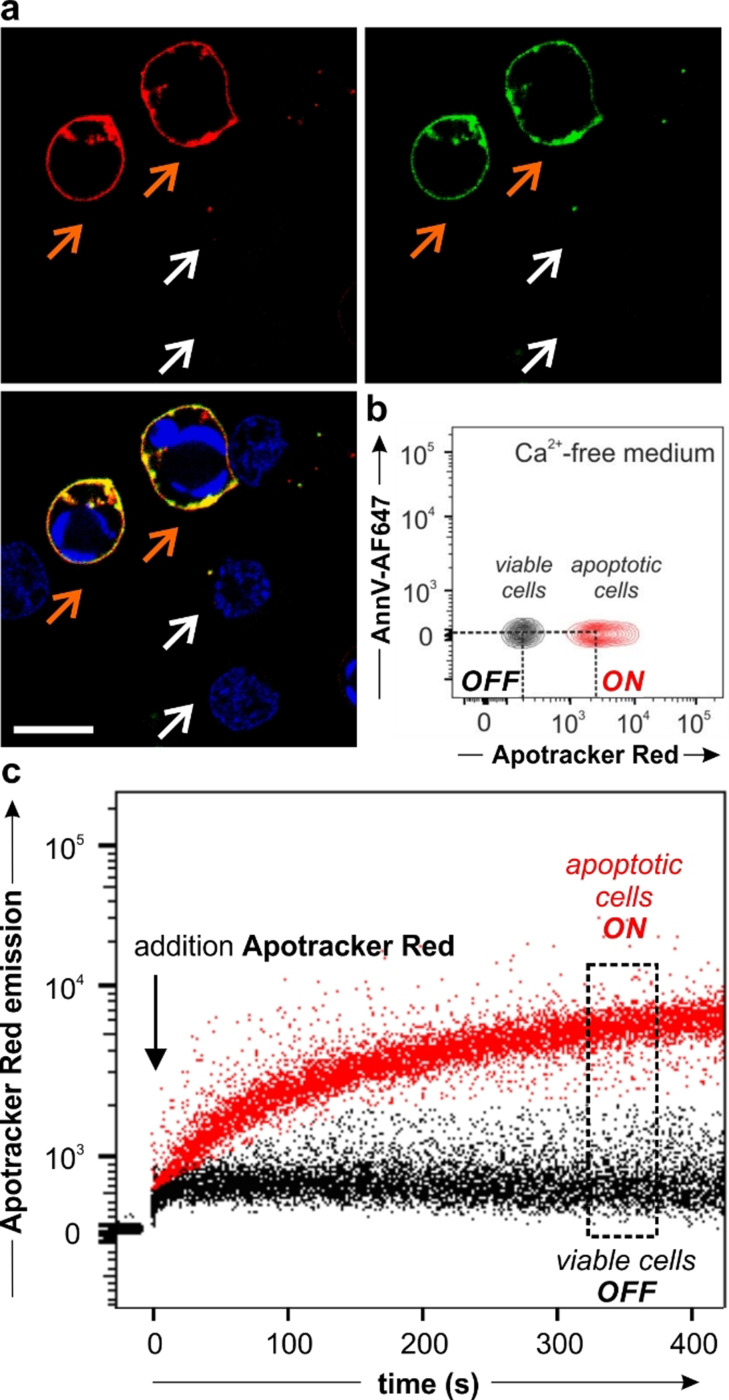
Apotracker Red selectively stains apoptotic cells in the presence of viable cells. a) Fluorescence microscopy images of Jurkat cells after treatment with 1 μM staurosporine and incubation with Apotracker Red (150 nM, red), Annexin V‐AF647 (5 nM, green) and Hoechst 33342 (7 μM, blue) for 10 min. Representative images from 3 independent experiments. White arrows identify viable cells and orange arrows identify apoptotic cells. Scale bar: 10 μm. Wavelengths (excitation/emission): Apotracker Red (561/610 nm), AnnexinV‐AF647 (647/670 nm), Hoechst (405/450 nm). b) Flow cytometric analysis of viable cells (black) and apoptotic human neutrophils (red) upon staining with Apotracker Red (150 nM) and AnnexinV‐AF647 (25 nM) in medium containing 2.5 mM EDTA. Representative plot from 3 independent experiments. c) Wash‐free time‐course analysis of Apotracker Red staining in mixtures of apoptotic (red) and viable (black) human neutrophils. The arrow features the addition of Apotracker Red (150 nM) to the cell mixture.

Next, we examined the utility of Apotracker Red for fluorescence imaging by performing confocal microscopy experiments in Jurkat cancer cells undergoing programmed cell death. In order to have a mixture of viable and apoptotic cells that would allow us to assess the selectivity of Apotracker Red in a single culture, we induced apoptosis in Jurkat cells by treatment with staurosporine, a kinase inhibitor that activates caspase‐dependent and caspase‐independent pathways of apoptosis.^[19]^ Following induction of apoptosis, we incubated the cells with Apotracker Red and imaged them directly under the confocal microscope. We used the generic nuclear dye Hoechst 33342 to detect all cells and Annexin V‐AlexaFluor647(AF647), a 36 kDa protein marker of PtdSer in the presence of Ca^2+^ ions, to determine the location of apoptotic cells. The images in Figure 2 a and Figure S5 show that Apotracker Red brightly labelled the surface of apoptotic cells with excellent signal‐to‐noise ratios, whereas it did not label viable cells that were counterstained with the nuclear dye but devoid of AnnexinV‐AF647 and Apotracker Red signals. These experiments were performed in the presence of 2 mM CaCl_2_, which is needed for annexins to bind PtdSer headgroups;^[20]^ however, we also confirmed that Apotracker Red was equally selective for apoptotic cells in the absence of Ca^2+^ (Figure 2 b; Figure S6). Even after chelation of free divalent cations using 2.5 mM EDTA (ethylenediaminetetraacetic acid), Apotracker Red showed robust staining of apoptotic cells, whereas Annexin V‐AF647 failed to bind under the same experimental conditions (Figure S7). The Ca^2+^‐independence of Apotracker Red represents an important advantage over annexins for cell‐based screens and in vivo studies of anticancer drugs, given that deregulated Ca^2 +^ signalling is linked to cancer hallmarks and that altered Ca^2+^ transporter protein expression has been associated with tumour development.[^20b^, ^21^]

In addition to showing good selectivity for target cells without the need for washing steps, fluorescent probes for screens must display favourable kinetics that enable rapid acquisition. To determine the labelling speed of Apotracker Red, we ran time‐course flow cytometry and simultaneously measured the fluorescence signals of Apotracker Red in apoptotic and viable cells (Figure 2 c). We observed that Apotracker Red could discern between apoptotic and viable cells within minutes and without any washing steps, reaching a plateau after 5–10 minutes. The results confirm that Apotracker Red selectively stains apoptotic cells in the presence of viable cells and displays optimal features (e.g., divalent cation independence, rapid binding, wash‐free labelling, red fluorescence emission) as a fluorescent probe for cell‐based in vitro assays and intravital imaging studies.

### Apotracker Red can be used for rapid screening of chemotherapeutic drugs in cancer cells of variable origin

**Figure 3:**
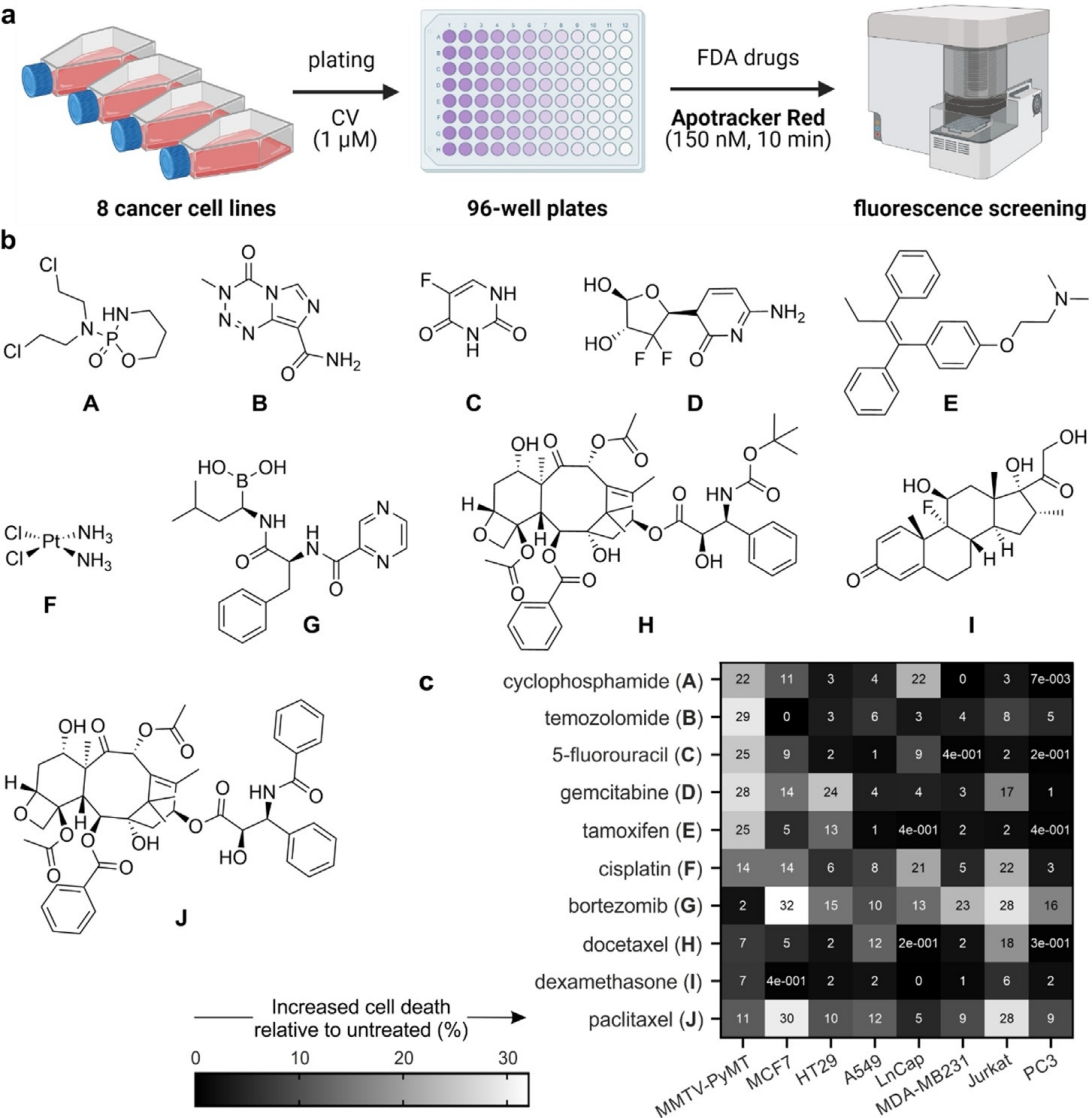
Apotracker Red enables rapid and wash‐free fluorescence screens of chemotherapeutic drugs in cancer cells of variable origin. a) Design of the fluorescence‐based assay for analysis of chemotherapeutic‐induced cancer cell death using Apotracker Red; CellTrace^TM^ Violet (CV). Wavelengths (excitation/emission): Apotracker Red (561/610 nm), CV (405/510 nm). b) Chemical structures of FDA‐approved small molecules. c) Heatmap of percent increase in Apotracker Red‐stained cells compared to untreated cells. Data presented as an average of 3 independent experiments.

Given the promising properties of Apotracker Red as a direct marker for apoptotic cancer cells, we examined its application for fluorescence‐based screens of small molecules with potential anticancer activity. We designed a screening platform in line with the standards of the National Cancer Institute‐60 (NCI‐60),^[22]^ where the evaluation of anticancer drug candidates consisted of two stages: 1) a single‐dose screen to identify the compounds inducing the most potent cell death as defined by Apotracker Red staining, and 2) a dose‐response study of shortlisted drugs to select those for in vivo studies. To corroborate the versatility of Apotracker Red, we built a screening platform including a total of 8 cancer cell lines of variable origin and pathogenesis: breast (MCF7, MDA‐MB231 and MMTV‐PyMT (mouse mammary tumour virus‐polyoma middle tumour‐antigen)^[23]^), lung (A549), prostate (PC3 and LnCap), blood cells (Jurkat) and colon (HT29). All cell lines were cultured under standard conditions and seeded with the fluorescent proliferation marker CellTrace^TM^ Violet (CV) in 96‐well plates (10–20×10^3^ cells/well) for high‐throughput flow cytometry. After drug treatment (i.e., drug addition plus 24 h incubation), Apotracker Red was incubated for 10 min and fluorescence readings were directly acquired without any additional processing steps (Figure 3 a). The low cost and simplicity of this platform are important advantages over current screening protocols that involve fixation and/or washing steps. Using this platform, we tested a collection of FDA‐approved drugs in all cancer cell lines and compared their ability to induce apoptosis using Apotracker Red as the reporter. Specifically, we included DNA alkylation agents (e.g., cyclophosphamide, cisplatin, 5‐fluorouracil, gemcitabine, temozolomide), proteasomal and mitotic inhibitors (e.g., bortezomib, docetaxel, paclitaxel) and antagonists of specific proteins (e.g., dexamethasone, tamoxifen; Figure 3 b).

**Figure 4:**
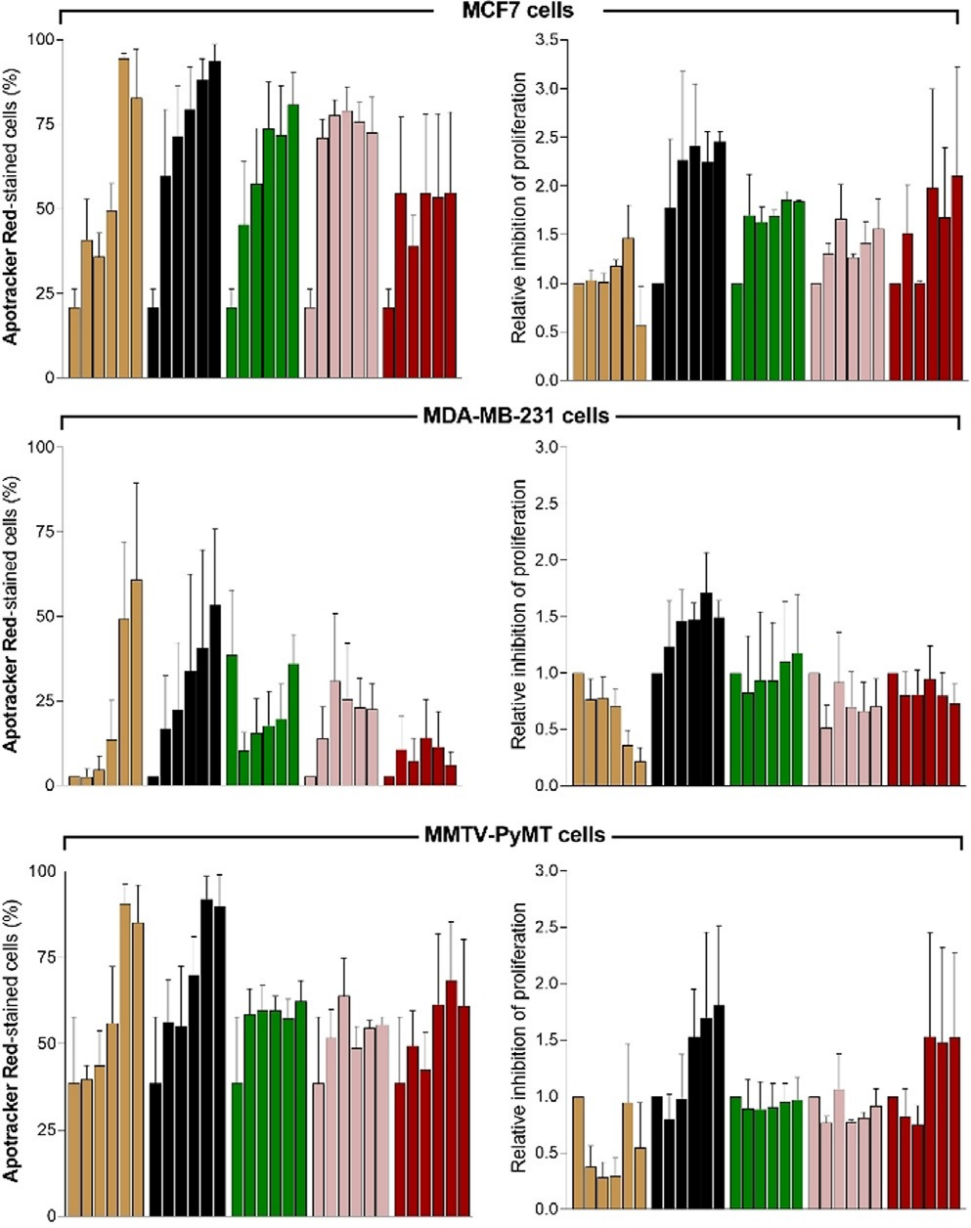
Apotracker Red detects dose‐dependent chemotherapeutic‐induced apoptosis in mouse and human breast cancer cell lines. Cells were plated with 1 μM CV, treated with increasing concentrations (0, 1, 3, 10, 30 and 100 μM) of selected drugs for 48 h, and stained with Apotracker Red (150 nM) before data acquisition. Drugs: tamoxifen (beige), cisplatin (black), 5‐fluorouracil (green), gemcitabine (pink) and cyclophosphamide (red). Data presented as mean±SEM of 3 independent experiments.

First, all drugs were tested in the 8 different cell lines to determine the percentages of Apotracker Red‐stained apoptotic cells compared to no treatment (Figure 3 c; Figure S8). Interestingly, we observed notable differences between drugs as well as between cell lines, indicating that not all cancer cells responded equally to the same compounds.^[24]^ Based upon the fluorescence staining of Apotracker Red (for apoptosis) and CV (for proliferation), we classified the drugs into 4 main groups: 1) gemcitabine and cisplatin induced apoptosis and decreased cell proliferation, 2) cyclophosphamide, tamoxifen, 5‐fluorouracil, paclitaxel and bortezomib induced apoptosis with little reduction of cell proliferation, 3) temozolomide and docetaxel inhibited cell proliferation but showed little or no induction of apoptosis, and 4) dexamethasone had no impact on proliferation or apoptosis. From this screen, we decided to select 5 drugs with potent pro‐apoptotic response in breast cancer, the most diagnosed cancer worldwide.^[25]^ From group 1, we selected both gemcitabine and cisplatin as the former elicited a strong response in MMTV‐PyMT cells and the latter showed a consistent response across mouse and human breast cancer cells. From group 2, we selected cyclophosphamide, tamoxifen, and 5‐fluorouracil as pro‐apoptotic molecules. Bortezomib and paclitaxel were discarded because they showed very minor effects on cell proliferation (Figure S8). All 5 drugs were tested at multiple concentrations (from 1 to 100 μM) and with incubation times up to 48 h to examine whether drugs could block cell proliferation at late stages (Figures S9–S13). The results from the dose‐dependency study highlighted tamoxifen and cisplatin as drugs with the highest percentages of Apotracker Red staining across all breast cancer cells, whereas gemcitabine, cyclophosphamide and 5‐fluorouracil showed weaker Apotracker Red‐staining in MDA‐MB‐231 cells (Figure 4). Cisplatin displayed the highest ratios of proliferation blockage; hence it was chosen for in vivo imaging studies. Altogether, these results prove the utility of Apotracker Red as a versatile tool for rapid fluorescence screening and mechanistic studies of chemotherapeutic‐induced cancer cell death.

### Intravital imaging of cisplatin‐induced cancer cell death in vivo in a mouse model of breast cancer

**Figure 5:**
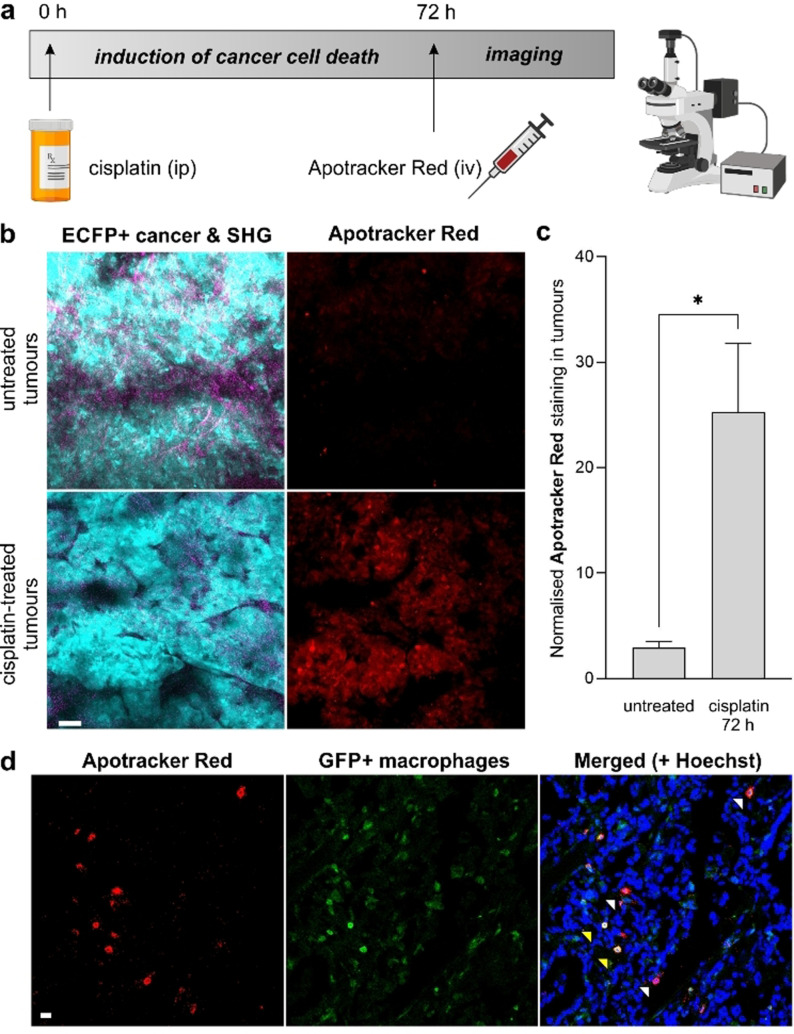
Apotracker Red is a marker for chemotherapy‐induced apoptosis of breast cancer cells in vivo. a) Experimental design for the in vivo treatment of mice bearing breast cancer tumours with cisplatin (10 μg g^−1^ intraperitoneally) followed by tail vein injection of 650 ng Apotracker Red on day 3 and intravital imaging and/or harvest of mammary gland tumours. b) Representative intravital images (from 2 independent experiments per group) of MMTV‐PyMT;ACTB‐ECFP tumours (cyan, excitation/emission wavelength: 890/480 nm), second harmonic generation (magenta, excitation/emission wavelength: 890/420 nm) and Apotracker Red (red, excitation/emission wavelength: 1070/620 nm). Scale bar: 50 μm. c) Quantification of cells stained with Apotracker Red in tumours from cisplatin‐treated and untreated mice. Apotracker Red signals were quantified in at least 5 tumour areas per mouse. Data shown as mean±SEM. *P* values were obtained from one‐way ANOVA using multiple comparisons (* for *p*<0.05). d) Ex vivo microscopy images of cisplatin‐treated mammary tumours highlighting cfms‐EGFP^+^ macrophages (green), nuclear staining (Hoechst 33342, blue) and apoptotic cells after in vivo administration of Apotracker Red (red). White and yellow arrowheads for tumour cells stained with Apotracker Red and macrophages. Scale bar: 10 μm. Representative images from 3 independent experiments.

Optical intravital imaging is increasingly used for the preclinical evaluation of anticancer drugs in vivo. Although imaging modalities like computed tomography, positron emission tomography and magnetic resonance imaging can help to visualise whole intact organs in a non‐invasive manner,^[26]^ they lack the spatiotemporal resolution needed to study dynamic interactions between individual tumour and stromal cells. This feature is critical to understand the mechanism of action of anticancer drugs and how they can affect interactions between cancer cells and immune cells in the tumour microenvironment.^[27]^ Intravital imaging heavily relies on the acquisition of fluorescence signals using multiphoton microscopy, which provides cellular resolution with deep tissue penetration (≈500 μm) and minimal photodamage.^[28]^ In the context of imaging drug‐induced tumour apoptosis, most studies rely on cancer cells expressing fluorescent caspase reporters, either in syngeneic models or after injection of transgenic cell lines. Because these reporters typically do not provide functional information about the state of cancer cells, we examined whether the administration of Apotracker Red to mouse models of breast cancer would enable intravital imaging of cisplatin‐induced tumour apoptosis in vivo. First, we analysed the chemical stability of Apotracker Red for in vivo studies by incubating the probe in mouse serum. The cyclic structure of Apotracker Red provides good proteolytic stability for up to 24 h (Figure S14), indicating good compatibility with tail vein administration for intravital imaging. We also confirmed that Apotracker Red did not cause cell toxicity at different working concentrations (Figure S15). Next, we investigated whether Apotracker Red could label cisplatin‐induced apoptosis in vivo in MMTV‐PyMT mice, a well‐established model of breast cancer that resembles the progressive stages of tumorigenesis in human luminal type B breast cancer.^[29]^ In the MMTV‐PyMT model, the polyoma middle T oncogene is expressed upon the mammary tumour virus promoter, which results in the development of mammary gland tumours. We employed mice expressing enhanced cyan fluorescent protein (ACTB‐ECFP) to grow mammary gland tumours for ≈6 weeks. When primary tumours measured around 0.5–1 cm in diameter, we administered cisplatin intraperitoneally (10 mg kg^−1^). Previous studies have reported strong chemotherapeutic action for cisplatin in vivo after 3 days;^[30]^ therefore, we injected Apotracker Red intravenously (650 ng per mouse) 72 h after the administration of cisplatin and used skin flap surgery to expose the mammary gland for intravital imaging using two‐photon microscopy (Figure 5 a).

We acquired time‐lapse microscopy images of both non‐treated and cisplatin‐treated mice that had received the same amount of Apotracker Red intravenously. In cisplatin‐treated mice, we observed strong red fluorescent signals indicating cancer cell death induced by the drug, whereas untreated mice were devoid of Apotracker Red fluorescence (Figure 5 b). Notably, Apotracker Red signals were mostly located in tissue areas with active remodelling (e.g., tumours) as indicated by second‐harmonic generation signals. Apotracker Red signals were stable for over 3 h of continuous multiphoton imaging, which features good photostability and suitability for longitudinal imaging studies (Supporting Information, Movies S1 and S2). We also used the fluorescence emission of Apotracker Red for the ex vivo characterisation of tumours to quantify the relative percentages of apoptotic cells, which featured significant differences between cisplatin‐treated and untreated mice (Figure 5 c). Importantly, these results indicate that the signals from Apotracker Red are not impaired by tissue processing steps and that they can be used to quantify chemotherapeutic action in vivo.

Finally, we assessed the in vivo selectivity of Apotracker Red in harvested tumours from mice expressing colony‐forming stimulating factor (cfms)‐EGFP macrophages, which had been treated with cisplatin and Apotracker Red. First, we did not observe changes in the number of macrophages found in both tumours (Figure S16). Moreover, fluorescence microscopy revealed little co‐localisation between the signals of Apotracker Red and EGFP‐positive macrophages (Figure 5 d).

This result suggests that 1) Apotracker Red is not largely taken up non‐specifically by phagocytic cells, and 2) the extent of efferocytosis (i.e., macrophage clearance of dead cells) during the imaging procedure is limited; hence, the majority of the Apotracker Red signals can be attributed to the presence of apoptotic cells in tumours. Finally, we performed intravital imaging under spinning disk fluorescence microscopy where we could follow the interactions between individual EGFP‐positive macrophages and Apotracker Red‐stained cancer cells over time (Figure S17, Movie S3). These results confirm the utility of Apotracker Red to study dynamic cellular events in the tumour microenvironment in vivo with high spatiotemporal resolution.

## Conclusion

In summary, we describe Apotracker Red as a new fluorescent probe for rapid and robust detection of apoptosis in vitro and in vivo. We have optimised a methodology for the SPPS of BODIPY‐labelled peptides using orthogonal Alloc protecting groups with improved recovery yields (>20 %) and minimum purification steps. Apotracker Red is the first fluorogenic probe to allow robust quantification of apoptotic cancer cells both in drug screens and in vivo in live tumours of breast cancer mouse models. Apotracker Red overcomes some limitations of existing probes (e.g., annexins) given its Ca^2+^ independence, two‐photon excitation, high chemical stability, and compatibility with wash‐free protocols. Furthermore, unlike protein‐based reagents, Apotracker Red can be scaled‐up in batches (>50 mg) to enable large in vitro screening assays and in vivo experiments. We have demonstrated the preferential binding of Apotracker Red to PtdSer‐expressing apoptotic cells and its application to high‐throughput screens using a broad range of cancer cell lines and FDA‐approved drugs. Furthermore, the suitability of Apotracker Red for intravital imaging can provide additional insights into the mode of action of anticancer therapeutics in vivo and to study the dynamics of drug action with high spatiotemporal resolution. All these features make Apotracker Red an optimal chemical tool to accelerate the identification and preclinical validation of anticancer therapeutics.

## Conflict of interest

The authors declare no conflict of interest.

## Supporting information

As a service to our authors and readers, this journal provides supporting information supplied by the authors. Such materials are peer reviewed and may be re‐organized for online delivery, but are not copy‐edited or typeset. Technical support issues arising from supporting information (other than missing files) should be addressed to the authors.

Supporting Information

Supporting Information

Supporting Information

Supporting Information
