# Supplementary material for: A Bivalent Activatable Fluorescent Probe for Screening and Intravital Imaging of Chemotherapy‐Induced Cancer Cell Death
Source: Angew Chem Weinheim Bergstr Ger. 2021 Dec 16;134(5):e202113020. doi: 10.1002/ange.202113020 (PMC10947113; doi:10.1002/ange.202113020)
Supplement: Supplementary file 1 — Supporting Information [file ANGE-134-0-s003.pdf]

Angewandte Chemie
Eine Zeitschrift der Gesellschaft Deutscher Chemiker

Supporting Information

A Bivalent Activatable Fluorescent Probe for Screening and Intravital Imaging of Chemotherapy-Induced Cancer Cell Death

*Nicole D. Barth, Lorena Mendive-Tapia, Ramon Subiros-Funosas, Ouldouz Ghashghaei, Rodolfo Lavilla, Laura Maiorino, Xue-Yan He, Ian Dransfield, Mikala Egeblad, and Marc Vendrell**

ange_202113020_sm_miscellaneous_information.pdf
ange_202113020_sm_Movie1.avi
ange_202113020_sm_Movie2.avi
ange_202113020_sm_Movie3.avi

Electronic Supporting Information

Table of Contents

1. Supplementary Figures
2. Experimental Methods
3. NMR Spectra
4. Supplementary Movie Legends
5. Supplementary References

Supplementary Figures

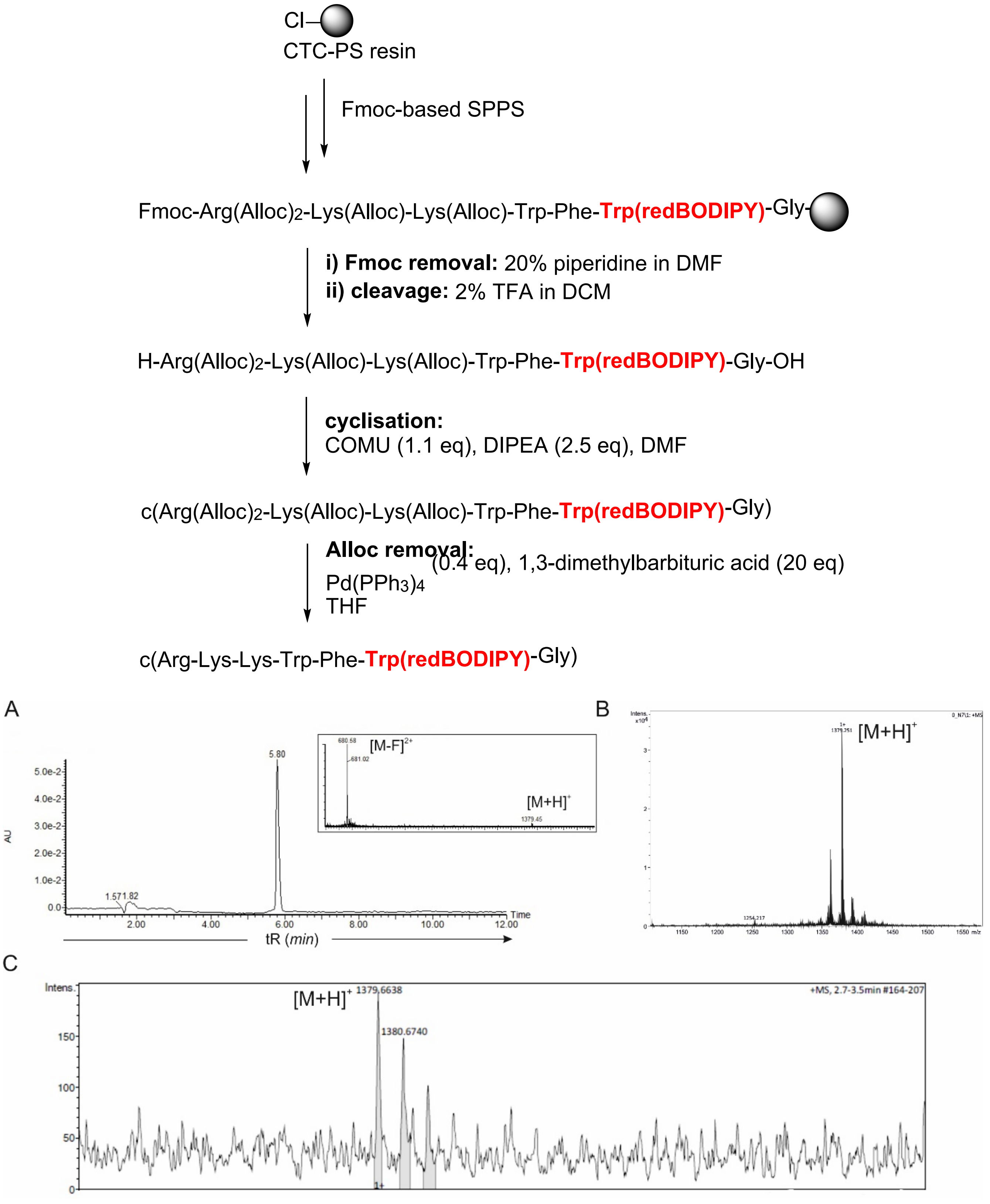

Figure S1. Synthetic scheme for the chemical peptide synthesis of **Apotracker Red** and chemical characterisation: A) HPLC trace (UV detection: 560 nm), B) MALDI spectrum, C) HRMS spectrum. $[M+H]^+$ calc. 1379.6642.

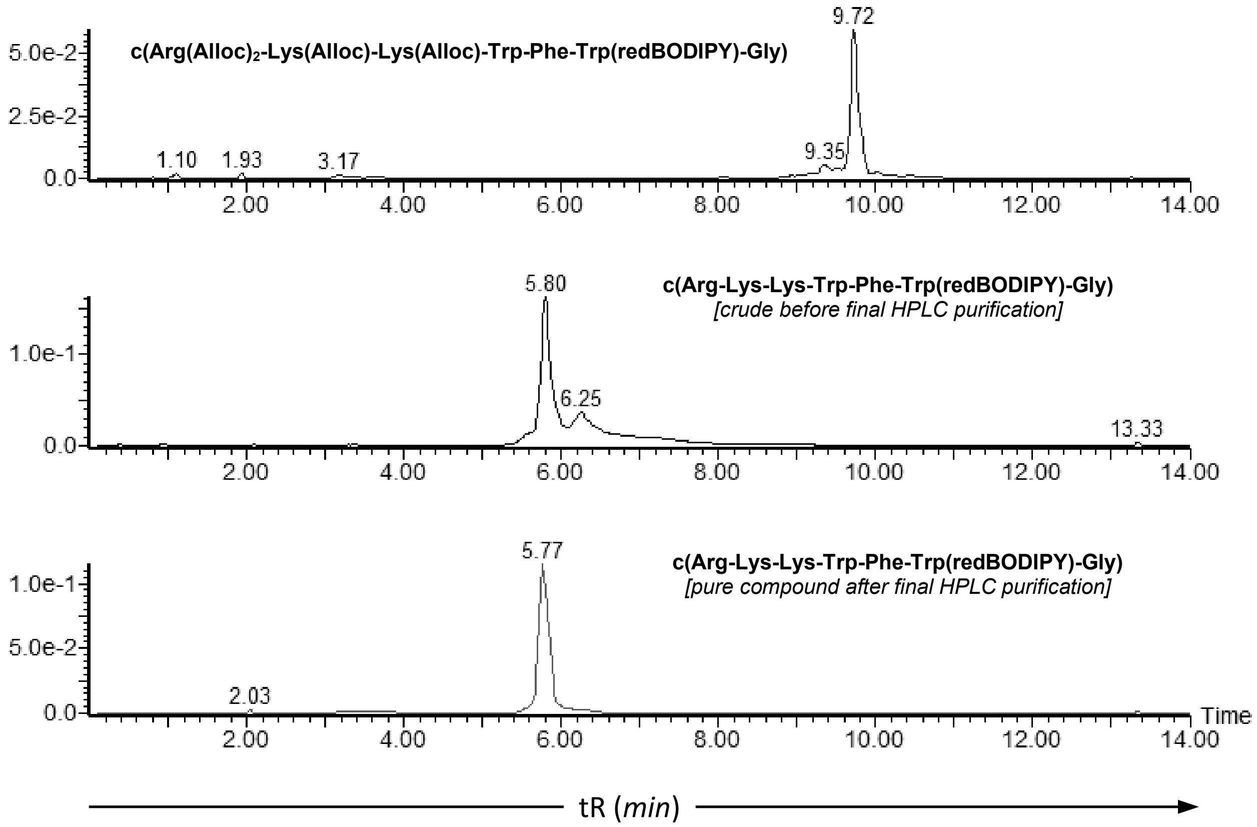

Figure S2. HPLC traces of **Apotracker Red** precursors after cyclisation (top panel), Alloc removal (middle panel) and final semi-preparative HPLC purification (bottom panel). UV detection: 560 nm.

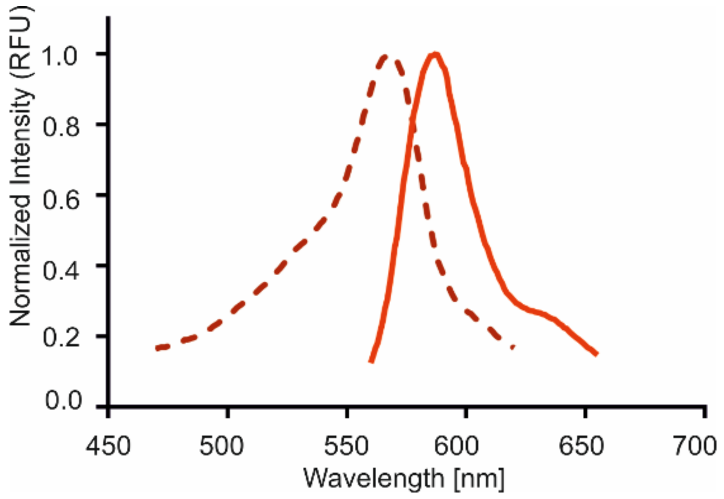

Figure S3. Absorbance and emission spectra of **Apotracker Red** (50 μM) in EtOH. λ_{exc}: 530 nm.

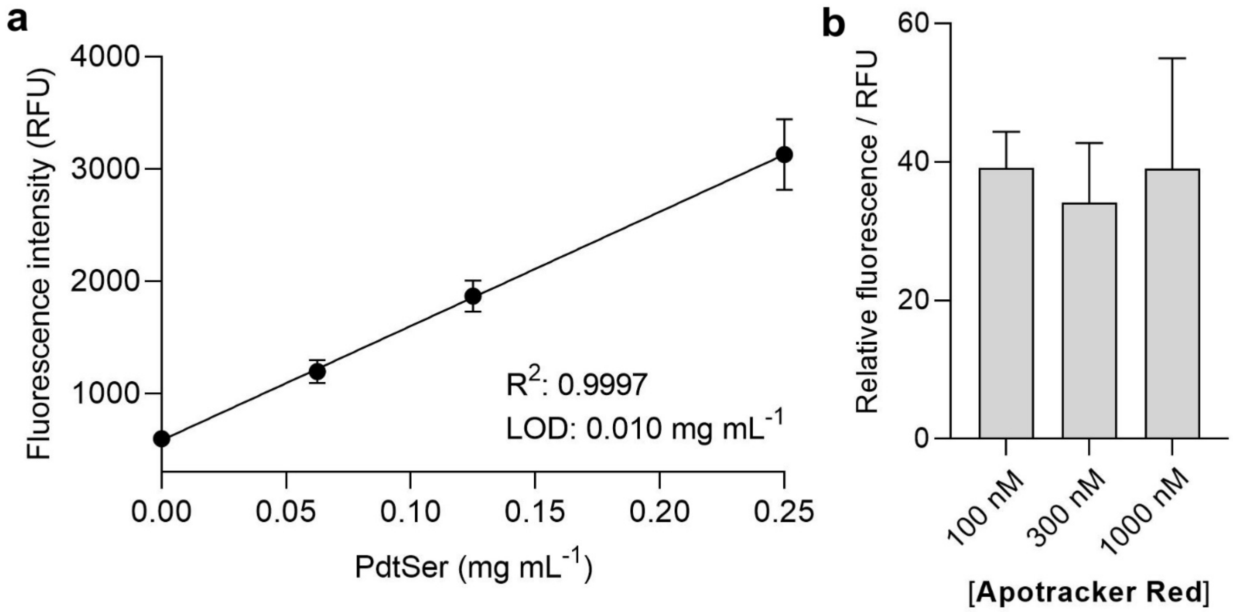

Figure S4. a) Linear correlation between fluorescence intensity of **Apotracker Red** (1 μM) and phosphatidylserine (PtdSer) concentration upon incubation for 40 min at 25 °C. Data presented as means ± SD (n=3). b) Relative fluorescence fold increase of apoptotic vs viable cells after incubation with **Apotracker Red** at different concentrations. Data as means ± SEM (n=4).

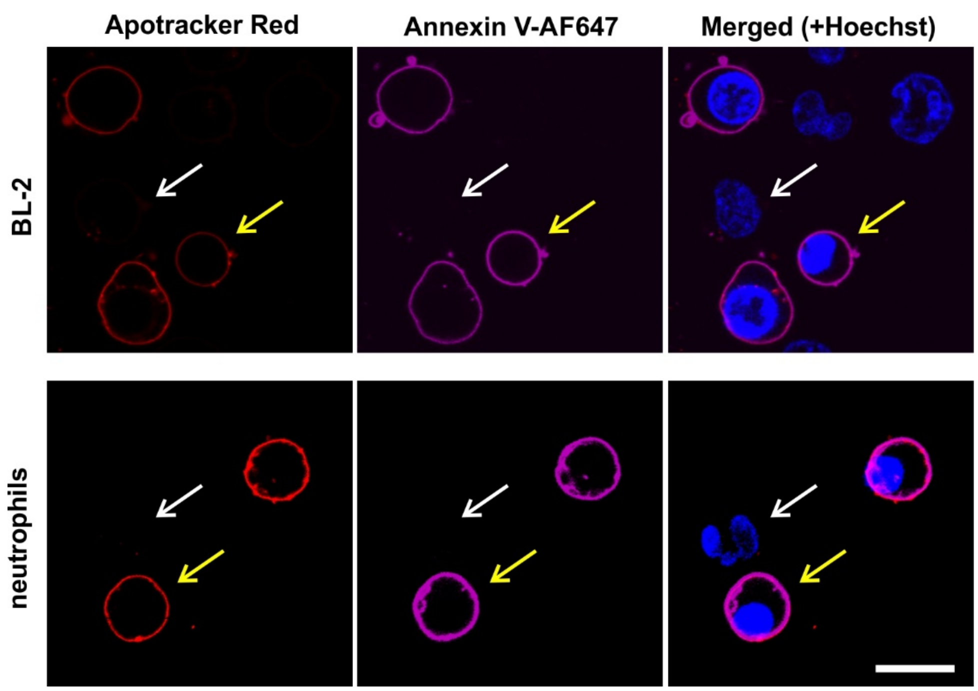

Figure S5. Representative microscopy images (from 3 independent experiments) of apoptotic (yellow arrows) and viable (white arrows) cell mixtures of Burkitt's lymphoma (BL-2) cells (top panel) and human primary neutrophils (bottom panel) after staining with 150 nM **Apotracker Red** (red), Annexin V-AF647 (25 nM, magenta) and Hoechst 33342 (7 μM, blue) in media containing 2 mM $CaCl_2$. (λ_{exc}: 405, 514, 633 nm: λ_{em}: 450, 600, 670 nm). S/N ratios (**Apotracker Red**) = 302 ± 2. Scale bar: 10 μm.

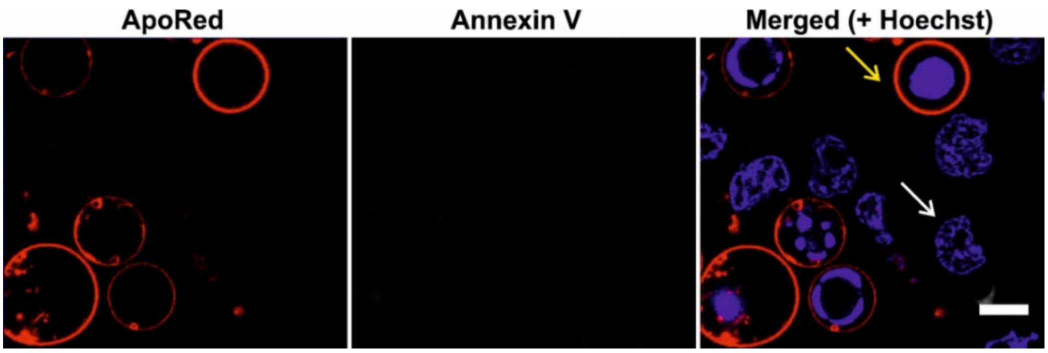

Figure S6. Representative microscopy images (from 3 independent experiments) of apoptotic (yellow arrow) and viable (white arrow) Jurkat T cells after incubation with staurosporine (1 μM). Cells were stained with 150 nM **Apotracker Red** (red), Annexin V-AF647 (5 nM, green), Hoechst 33342 (7 μM, blue) in Ca^{2+}-free media. (λ_{exc}: 405, 514, 633 nm: λ_{em}: 450, 600, 670 nm). Scale bar: 10 μm.

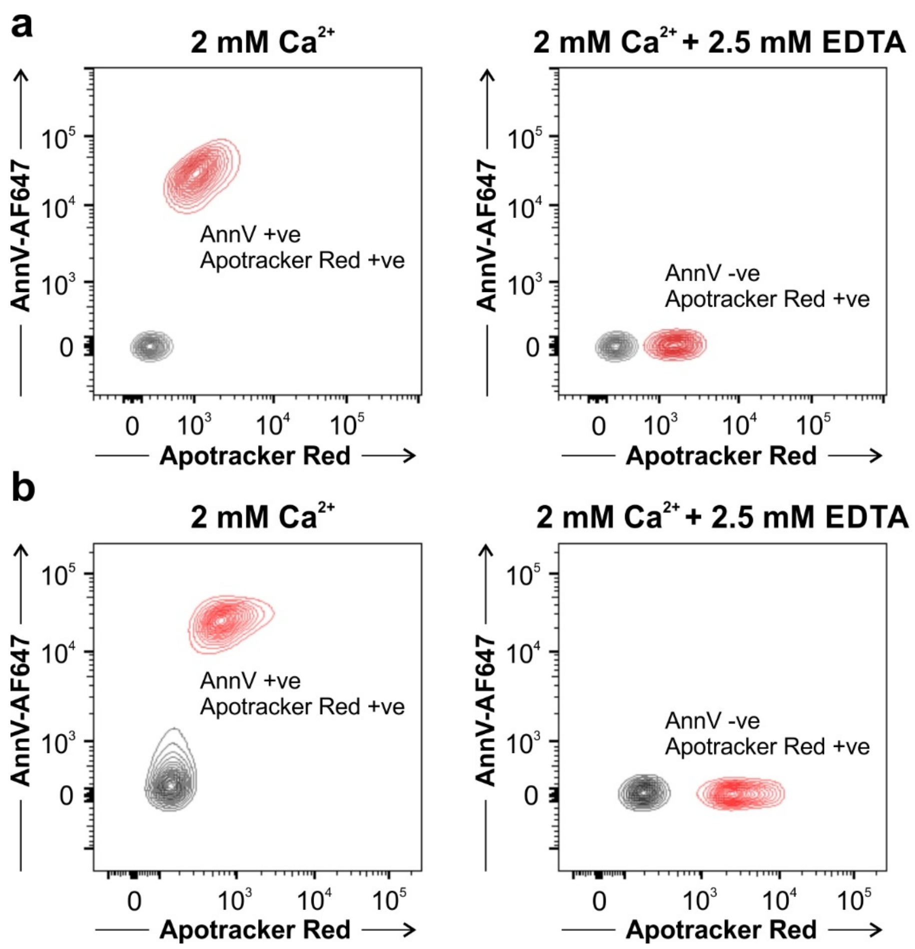

Figure S7. Divalent cation-independent staining of apoptotic cells using **Apotracker Red**. Representative contour plots (from 3 independent experiments) of Annexin V-AF647 (25 nM, left) and **Apotracker Red** (150 nM, right) labelling of viable cells (grey) and apoptotic cells (red) (a: primary human neutrophils, b: Jurkat T cells) in media including 2 mM $CaCl_2$ with or without 2.5 mM EDTA. Wavelengths (exc/em): AnnexinV-AF647 (647/670 nm), **Apotracker Red** (561/610 nm).

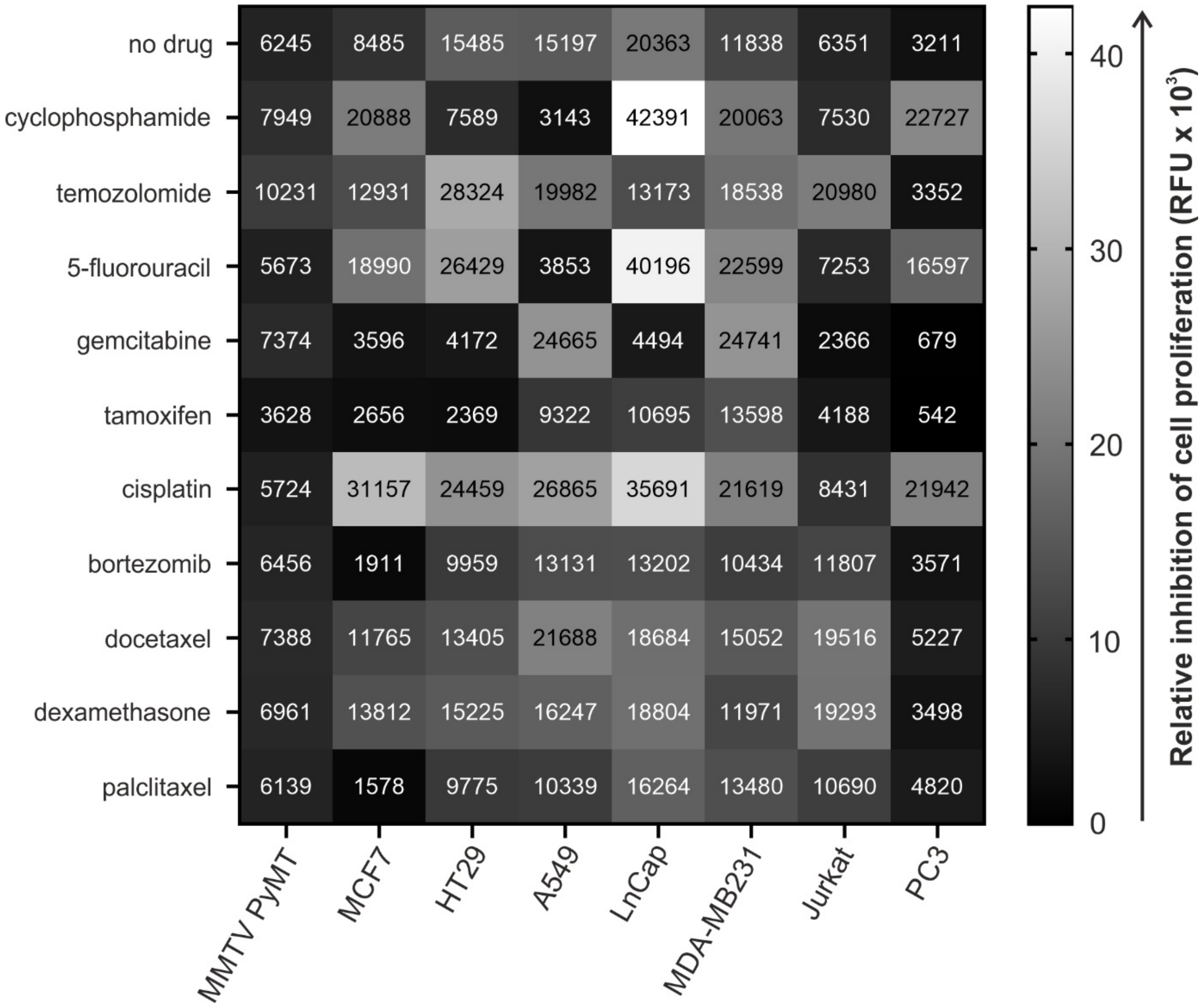

Figure S8. Heatmap of relative inhibition of cell proliferation (as indicated by staining of CellTrace^{TM} Violet) after treatment of cancer cell lines with different drugs. Data presented as an average of 3 independent experiments. Cells were stained on day 0 with CellTrace^{TM} Violet (1 μM) and mean fluorescence intensities were determined on the 5L LSR after 24 h of treatment with the different drugs.

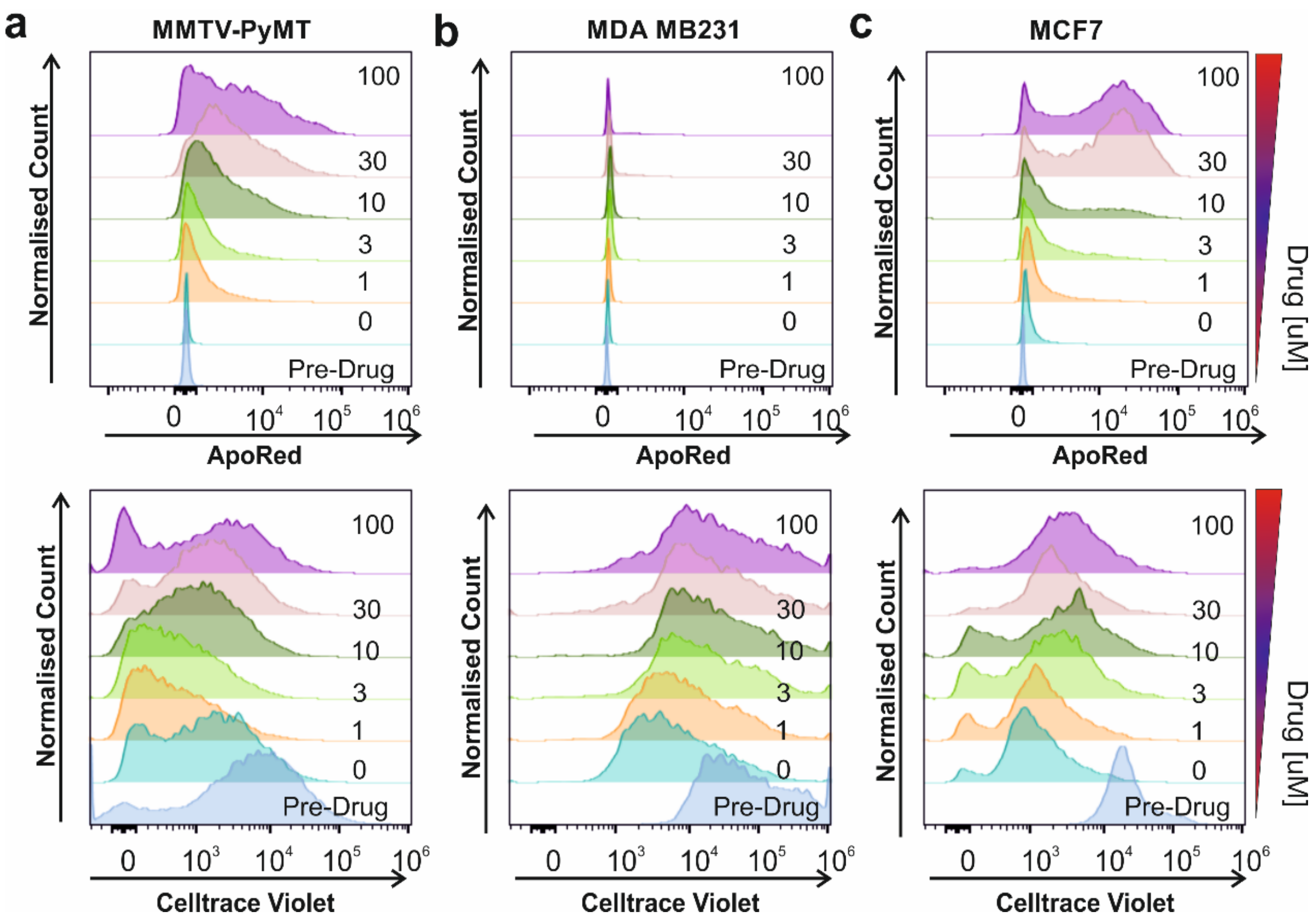

Figure S9. Flow cytometry analysis of cisplatin in different breast cancer cell lines. Representative flow cytometric plots (from three independent experiments) of MMTV-PyMT cells (a), MDA-MB231 cells (b) and MCF7 cells (c) after treatment with increasing concentrations of cisplatin for 48 h. Cells were stained on day 0 with CellTrace™ Violet (1 μM) and with **Apotracker Red** (150 nM) before data acquisition.

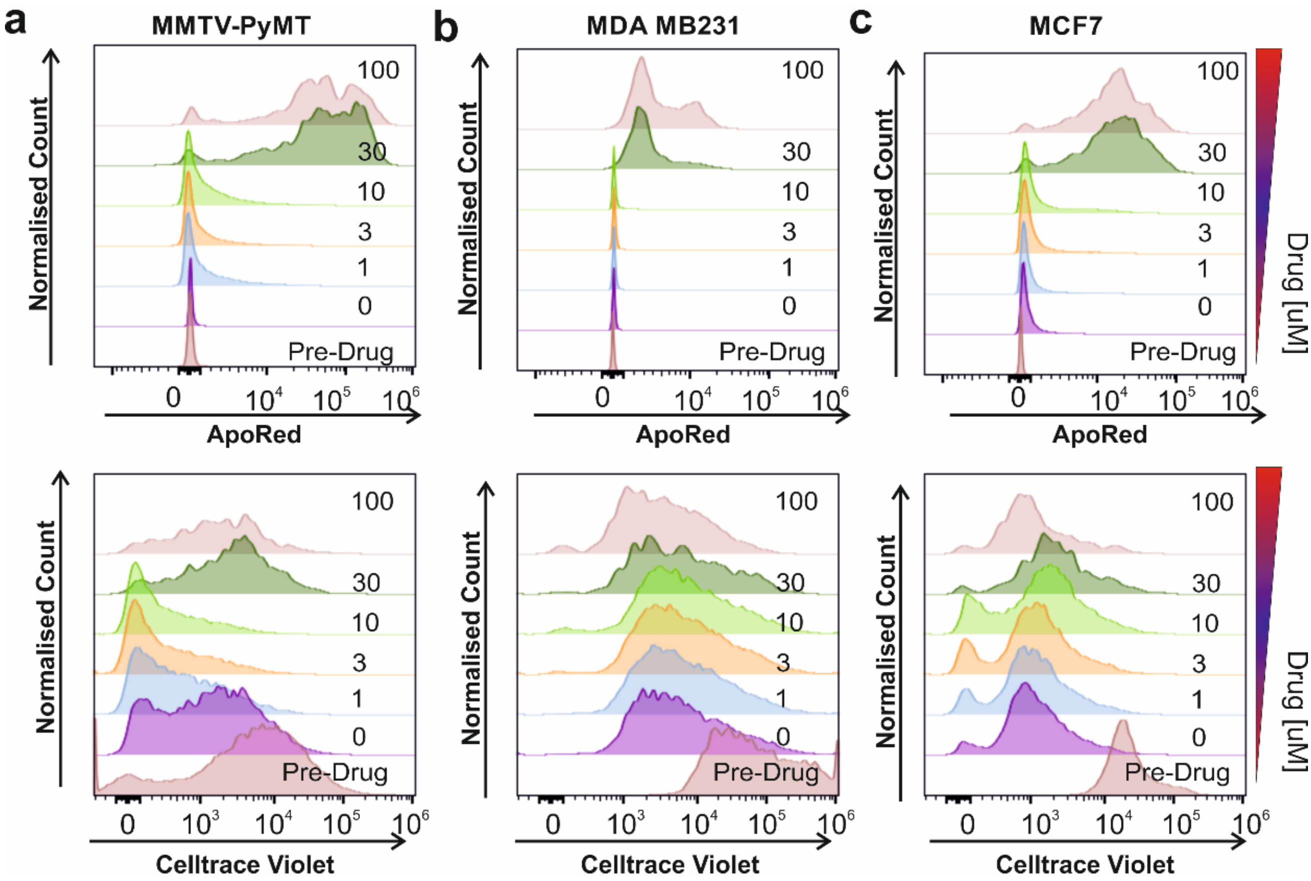

Figure S10. Flow cytometry analysis of tamoxifen in different breast cancer cell lines. Representative flow cytometric plots (from three independent experiments) of MMTV-PyMT cells (a), MDA-MB231 cells (b) and MCF7 cells (c) after treatment with increasing concentrations of tamoxifen for 48 h. Cells were stained on day 0 with CellTrace^{TM} Violet (1 μM) and with **Apotracker Red** (150 nM) before data acquisition.

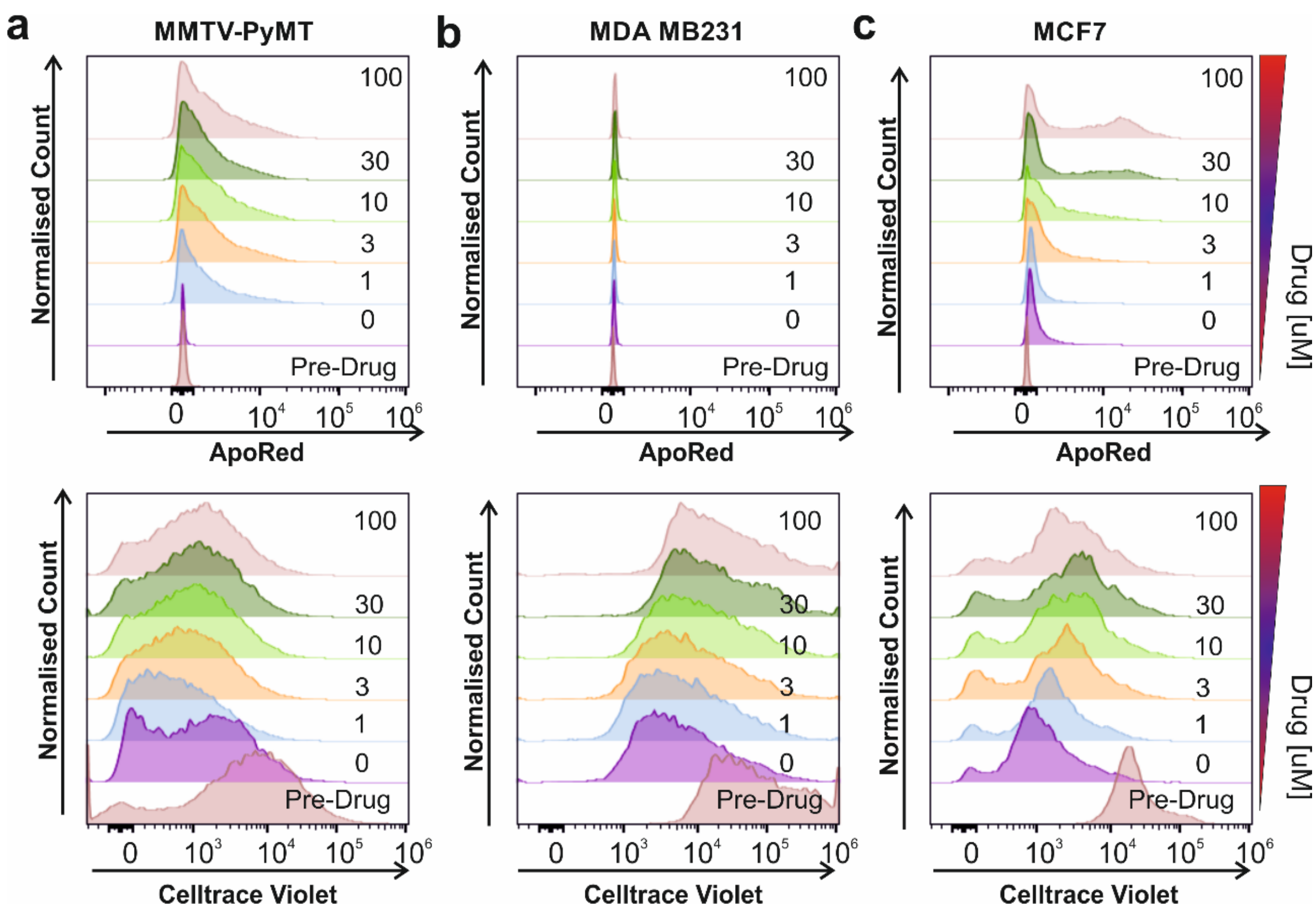

Figure S11. Flow cytometry analysis of gemcitabine in different breast cancer cell lines. Representative flow cytometric plots (from three independent experiments) of MMTV-PyMT cells (a), MDA-MB231 cells (b) and MCF7 cells (c) after treatment with increasing concentrations of gemcitabine for 48 h. Cells were stained on day 0 with CellTrace™ Violet (1 μM) and with **Apotracker Red** (150 nM) before data acquisition.

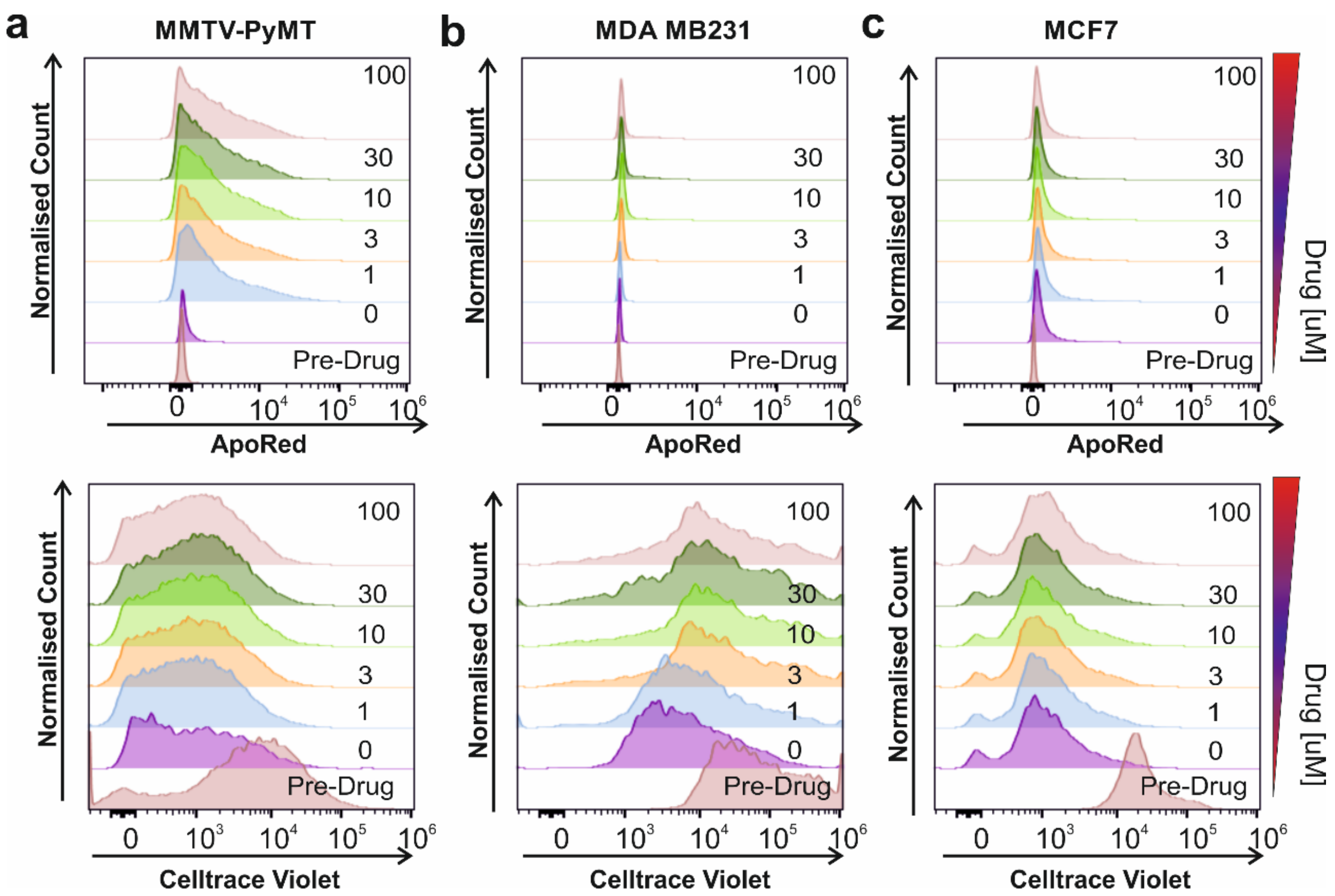

Figure S12. Flow cytometry analysis of 5-fluorouracil in different breast cancer cell lines. Representative flow cytometric plots (from three independent experiments) of MMTV-PyMT cells (a), MDA-MB231 cells (b) and MCF7 cells (c) after treatment with increasing concentrations of 5-fluorouracil for 48 h. Cells were stained on day 0 with CellTrace™ Violet (1 μM) and with **Apotracker Red** (150 nM) before data acquisition.

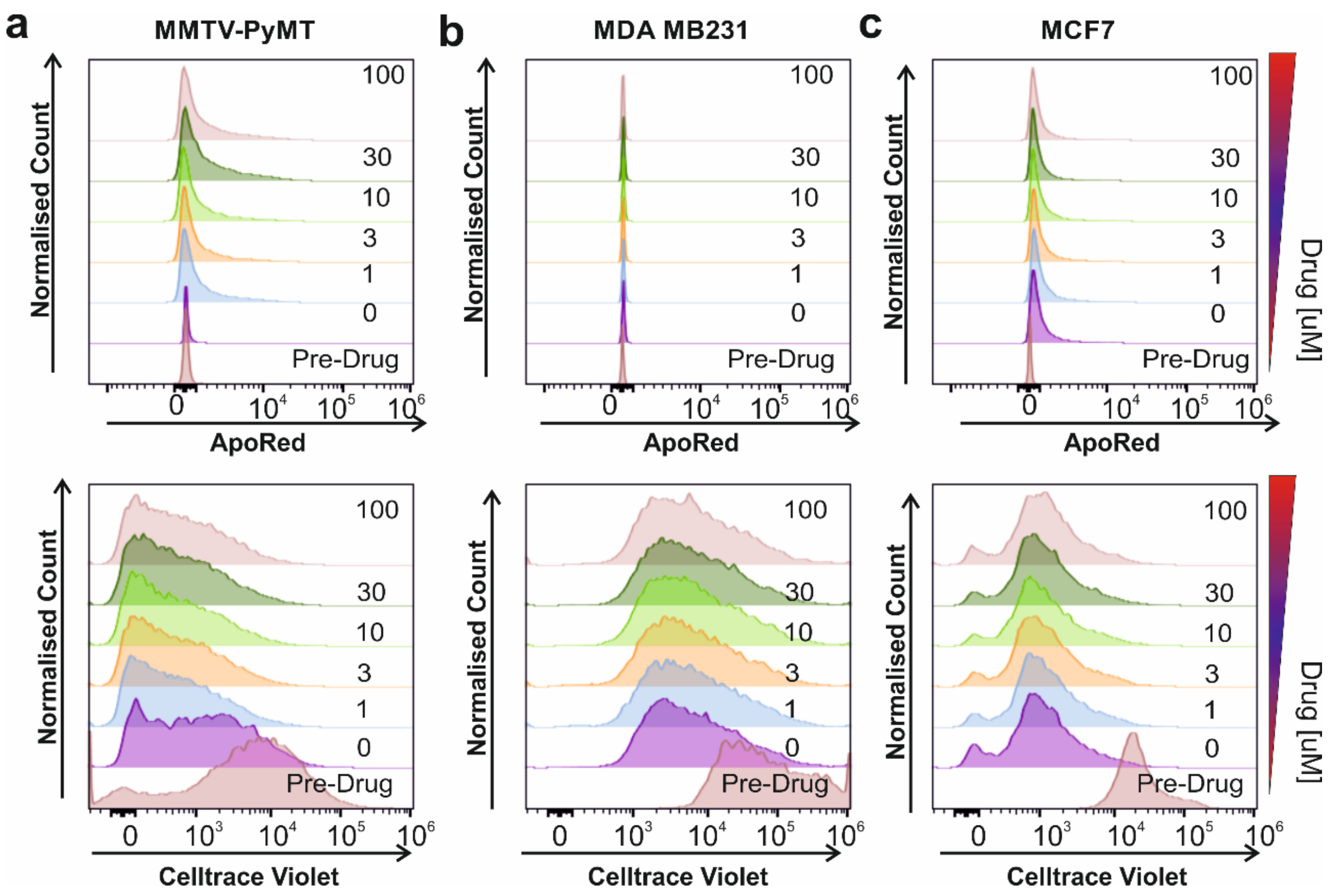

Figure S13. Flow cytometry analysis of cyclophosphamide in different breast cancer cell lines. Representative flow cytometric plots (from three independent experiments) of MMTV-PyMT cells (a), MDA-MB231 cells (b) and MCF7 cells (c) after treatment with increasing concentrations of cyclophosphamide for 48 h. Cells were stained on day 0 with CellTrace^{TM} Violet (1 μM) and with **Apotracker Red** (150 nM) before data acquisition.

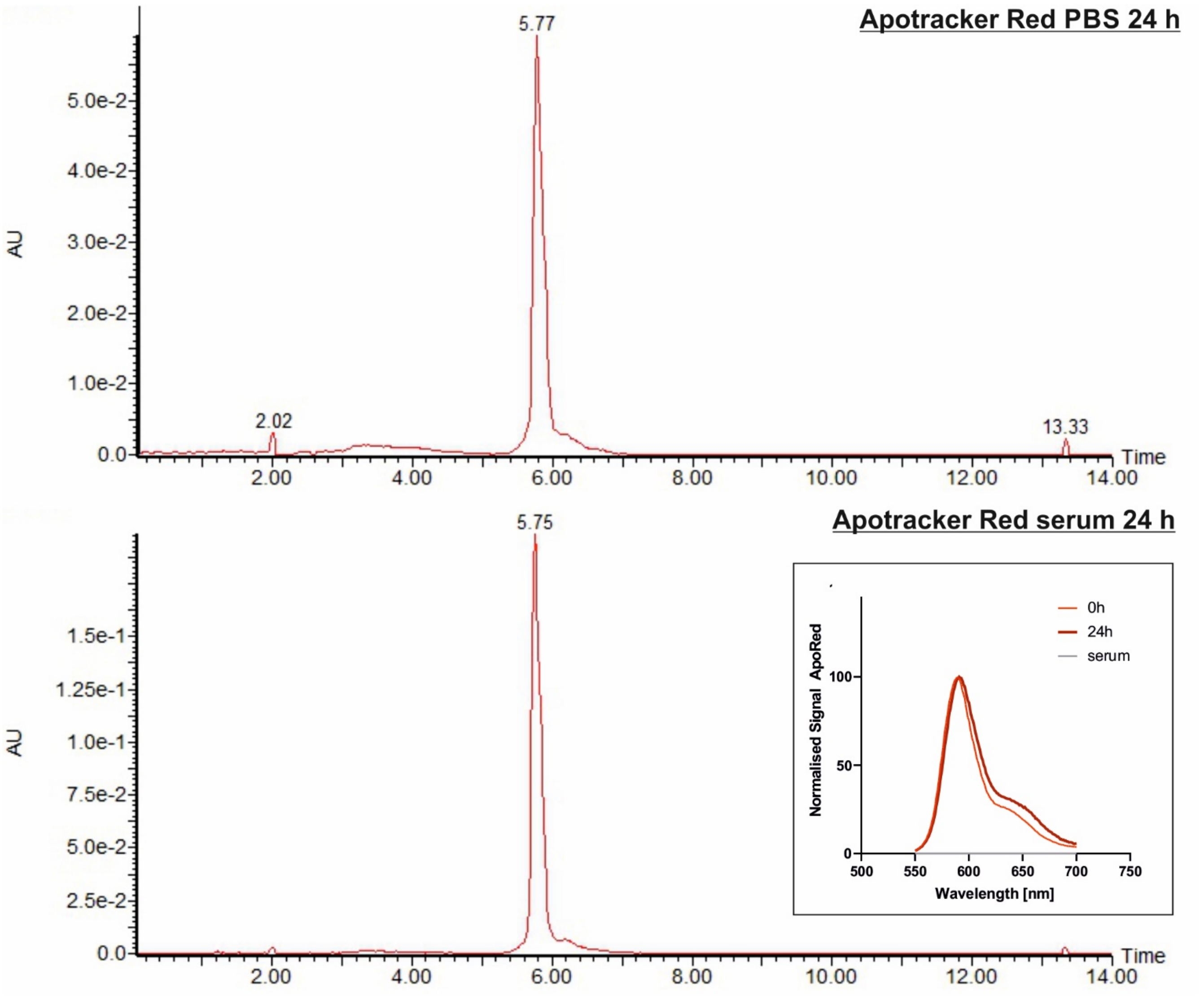

Figure S14. Stability analysis of **Apotracker Red** in mouse serum. HPLC traces of **Apotracker Red** (25 µM) after incubation in PBS buffer or mouse serum for 24 h at 37 °C. Inset shows fluorescence emission of **Apotracker Red** before and after incubation in mouse serum and excitation at 520 nm.

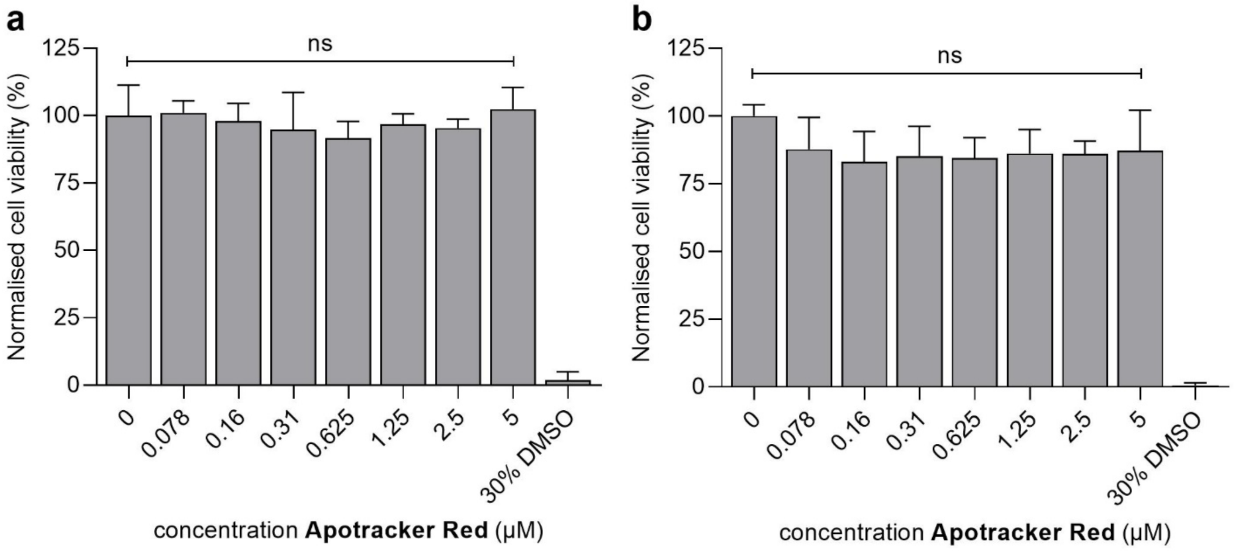

Figure S15. Cell viability experiments (TACS® MTT cell proliferation assay) after incubation of **Apotracker Red** in breast cancer cell lines (a: MCF7 cells, b: MDA-MB-231 cells) at different concentrations for 3 h. Data presented as means ± SD (n=5). P values obtained from one-way ANOVA using multiple comparisons (ns for p>0.05).

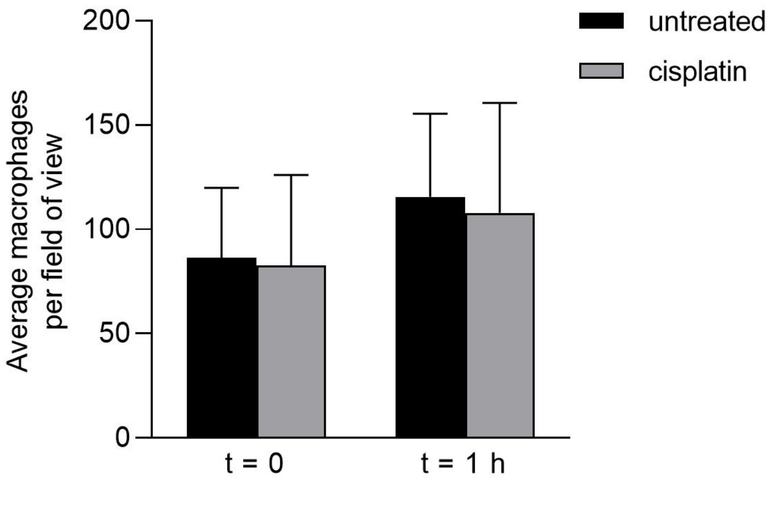

Figure S16. Quantification of EGFP^{+} macrophages in tumours from untreated and cisplatin-treated mice by time-lapse imaging on a spinning disk confocal microscope (λ_{exc}: 488 nm). Data presented as means ± SD of 8 random areas in 2-3 mice per group (untreated or treated with 10 µg g^{-1} cisplatin for 72 h).

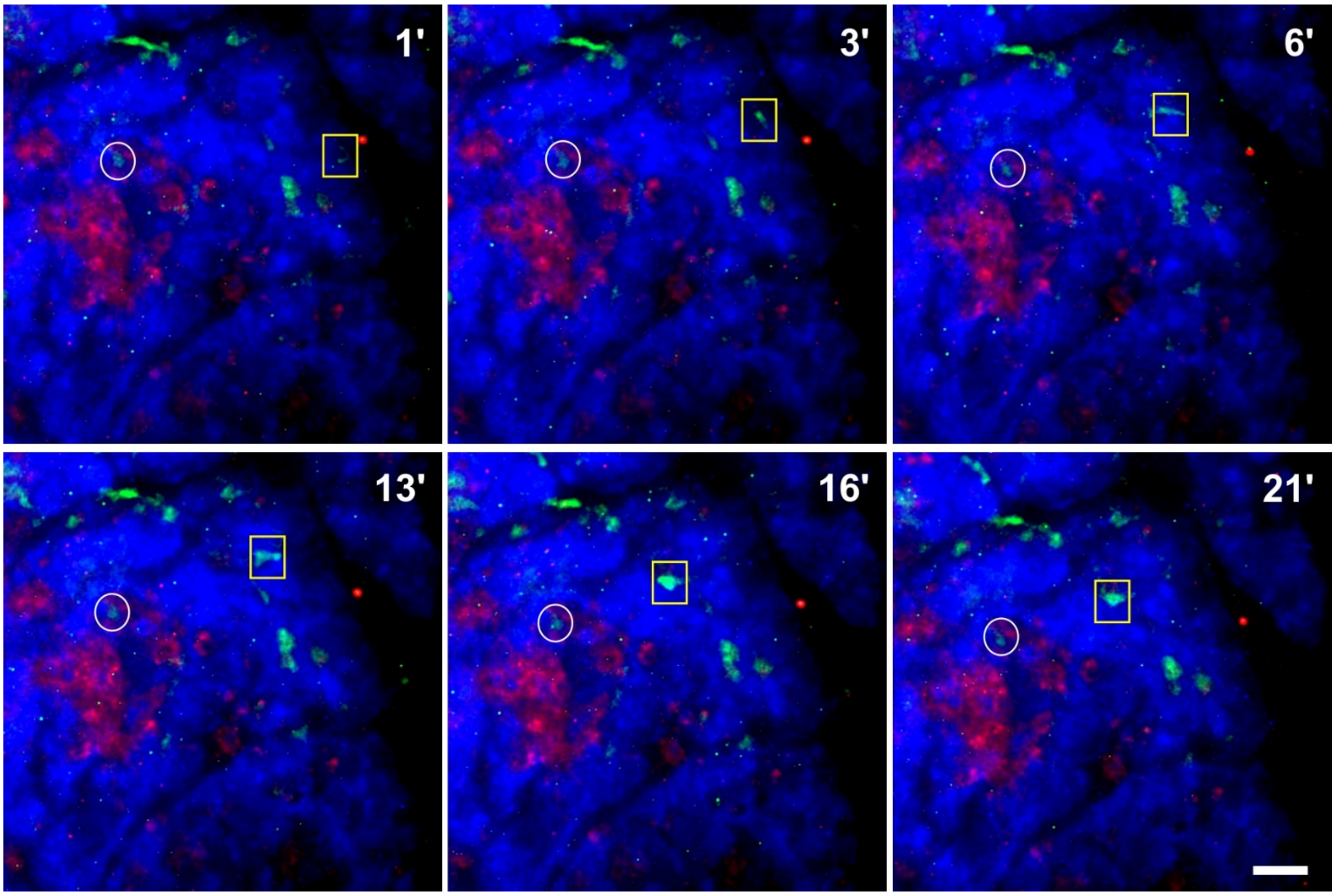

Figure S17. Time-course intravital images of cisplatin-treated mammary tumours highlighting ECFP$^+$ cancer cells (blue), EGFP$^+$ macrophages (green) and apoptotic cells after *in vivo* administration of **Apotracker Red** (red). Yellow squares highlight how a recruited macrophage moves towards apoptotic tumour cells, whereas the white circles feature a macrophage in close proximity to **Apotracker Red**-stained tumour cells (Supplementary Movie 3). Scale bar: 30 μm.

Experimental Methods

General experimental information.

Commercially available reactants were used without further purification. Thin-layer chromatography was conducted on Merck silica gel 60 F254 sheets and visualised by UV (254 nm and 365 nm). Reactions were monitored by HPLC-MS analysis using a HPLC Waters Alliance HT comprising a Kinetex C18 column (5 µm, 100 Å, 150 x 4.6 mm), a diode array and a MS detector configured with an electrospray ionisation source (Micromass ZQ4000). Unless otherwise indicated, A: H_2O (0.1% HCOOH) and B: CH_3CN (0.1% HCOOH) were used as eluents in a gradient from 0-100% B over 8 min with flow 1 mL min^{-1}. Data acquisition was performed with MassLynx software. **Apotracker Red** was manually synthesized in 2 mL polystyrene syringes fitted with porous polyethylene discs, using conventional SPPS protocols at r.t. and protected from light. Solvents, excess of reagents and soluble byproducts were removed by suction. **Apotracker Red** purification was conducted in a semi-preparative Agilent HPLC consisting of a 1220 Infinity II autosampler and a 1260 Infinity II detector. A Kinetex 150 × 21.2 mm (5 µm) C18 column was used, together with H_2O (0.1% HCOOH) and CH_3CN (0.1% HCOOH) as eluents and a flow rate of 8 mL min^{-1}. UV-Vis absorption measurements were performed using Thermo Scientific Nanodrop 1000 Spectrophotometer. NMR spectra were recorded on a 500 MHz spectrometer. Chemical shifts (δ) are reported in ppm. Multiplicities are referred by the following abbreviations: s = singlet, d = doublet, dd = double doublet and m = multiplet. HRMS (ESI-positive) were obtained with a Bruker ESI Micro-TOF mass spectrometer. MALDI analysis was performed on a Bruker UltrafleXtreme MALDI TOF-TOF mass spectrometer.

Chemical synthesis

Fmoc-Trp(redBODIPY)-OH. The synthesis was performed according to reported procedure.[1]

^{1}H NMR (500 MHz, $CDCl_3$, data for the major rotamer): δ 8.25 (s, 1H), 7.98 (m, 1H), 7.93 – 7.83 (m, 1H), 7.80 – 7.56 (m, 4H), 7.55 – 7.28 (m, 7H), 7.27 – 7.11 (m, 4H), 6.86 – 6.77 (m, 1H), 6.56 (d, J = 4.4 Hz, 1H), 6.48 (dd, J = 12.9, 4.5 Hz, 1H), 6.04 (d, J = 14.7 Hz, 1H), 5.19 (m, 1H), 4.63 (m, 1H), 4.30 – 4.12 (m, 2H), 4.08 (m, 1H), 3.59 – 3.38 (m, 2H), 2.61 (m, 3H), 2.54 – 2.42 (m, 3H), 1.54 (m, 3H) ppm.

^{13}C NMR (126 MHz, $CDCl_3$): δ 175.0, 163.0, 159.1, 155.9, 148.8, 144.0, 143.9, 143.8, 141.4, 139.6, 137.0, 136.0, 135.5, 135.2, 135.1, 133.2, 132.4, 132.2, 130.7, 129.4, 129.2, 129.1, 129.0, 128.9 (two peaks), 128.7, 127.8, 127.5, 127.2, 127.2, 125.2, 123.1, 122.7, 120.6, 120.1, 119.3, 119.2, 118.8, 111.2, 108.1, 108.0, 67.2, 54.7, 47.2, 29.8, 15.6, 15.2, 14.9 ppm.

HPLC-MS (ESI-): t_R: 6.0 min (gradient from 70-100% B, 95% purity).

HRMS (ESI+) m/z calcd. for $C_{48}H_{40}BF_2N_4O_4S$ $[M+H]^+$: 817.2826, found: 817.2783.

Apotracker Red. The synthesis was carried out using 20.1 mg of 2-chlorotrityl polystyrene resin (0.7 mmol g^{-1}). Fmoc-Gly-OH (1.0 eq) was loaded onto the resin (1.0 eq.) using DIPEA (3.0 eq) in DCM for 10 min followed by additional DIPEA (7.0 eq) for an extra 40 min. MeOH (0.8 µL mg^{-1} resin) was added to cap remaining trityl groups. The resin was then filtered and washed with DCM (5 × 1 min) and DMF (5 × 1 min). The loading of the resin was determined by measuring the absorbance of piperidine-dibenzofulvene adduct at 290 nm using Nanodrop. After the Fmoc groups were removed with 20% piperidine in DMF (1 × 1 min, 2 × 5 min), the resin was washed with DMF (4 × 1 min), DCM (3 × 1 min) and DMF (4 × 1 min). Unless otherwise stated, each amino acid was double coupled using Fmoc-AA-OH (4.0 eq), COMU (4.0 eq), OxymaPure (4.0 eq) and DIPEA (8.0 eq) in DMF for 1 h and 1 min pre-activation. Fmoc-Arg$(Alloc)_2$-OH (3.0 eq) was subjected to an extra coupling with PyOxim (3.0 eq) and DIPEA (6.0 eq) in DMF for 2 h. Fmoc-Trp(redBODIPY)-OH (1.1 eq) was incorporated with COMU (1.1 eq), OxymaPure (1.1 eq) and DIPEA (2.0 eq) in DMF for 2 h. The resin was then washed with DMF (5 × 1 min), DCM (5 × 1 min), and DMF (5 × 1 min). Before the next coupling cycle, Fmoc groups were removed as

described above. Once the sequence was fully elongated, the peptide was cleaved from the resin using 2% TFA in DCM (5 × 1 min) and washed with DCM (5 × 1 min). The combined filtrates were collected into a round bottom flask containing DCM (20 mL). Solvent was evaporated under reduced pressure and the remaining residue was washed with cold Et_2O, dissolved in CH_3CN:H_2O (1:1) and lyophilised to afford a dark purple solid (19.8 mg). Cleaved peptide (1.0 eq) was then dissolved in DMF (0.055 M) with COMU (1.1 eq) and DIEA (2.5 eq) and the resulting mixture was stirred for 1 h at r.t. The solution was diluted in DCM (10 mL) and washed with H_2O (2 x 10 mL), back extracting the aqueous layers with DCM. Organic layers were combined and after removal of the solvent under reduced pressure, the crude was dissolved in CH_3CN:H_2O (1:1) and lyophilised to afford a dark purple solid corresponding to the Alloc-protected **Apotracker Red** (16.2 mg). The crude macrocycle (1.0 eq) was deprotected with 1,3-dimethylbarbituric acid (20.0 eq) and $Pd(PPh_3)_4$ (0.4 eq) in THF (0.052 M) for 1 h. Purification was conducted by semi-preparative HPLC using a 0-100% gradient over 25 min, with detection at 560 nm. Pure fractions were collected and lyophilised to afford pure **Apotracker Red** as a purple solid (4.0 mg, 2.9 µmol, 22% overall yield).

HPLC-MS (ESI+): t_R: 5.8 min (gradient from 0-100% B, 97% purity).

HRMS (ESI+) m/z calcd. for $C_{73}H_{86}BF_2N_{16}O_7S$ $[M+H]^+$: 1379.6642, found 1379.6695.

MALDI (m/z): $[M+H]^+$ found 1379.6681.

Spectral characterisation. Spectroscopic data was recorded on Cytation 3 (Biotek). **Apotracker Red** was dissolved at 5 mM in DMSO and diluted to the indicated concentrations. Absorbance spectra were recorded on 96-well plates. To determine the relative fluorescence quantum yields, Rhodamine 101 was used as a reference (QY = 0.96 in MeOH).[2]

Stability of Apotracker Red in mouse serum. Mouse blood was drawn from 8 week-old C57BL/6 mice into 10% 0.5 M EDTA. Cells were removed by centrifugation for 10 min, 300 g at

r.t., followed by separation from erythrocytes by centrifugation for 10 min, 2,000 g at r.t. Sera were transferred into new tubes and stored at -20°C until use. 50 μM **Apotracker Red** in PBS or in serum were incubated for 24 h at 37 °C on a vertical shaker at 500 rpm. As controls, serum without peptide and freshly aliquoted 50 μM **Apotracker Red** in mouse serum were used. Fluorescence spectra were recorded after excitation at 520 nm. For HPLC measurements, proteins were precipitated by addition of CH_3CN and centrifugation at 300 g for 5 min at r.t. and the resulting supernatants were injected.

Lipid layer assays. Cardiolipin (CL), phosphatidylglycerol (PG) and phosphatidic acid (PA) were purchased from Stratech Scientific Ltd. Phosphatidylserine (PtdSer) and phosphatidylcholine (PC) were obtained from Sigma Aldrich. All lipids were dissolved at 1 mg mL^{-1} in dry EtOH by vigorous vortexing for 5 min followed by sonication for 20 min. 100 μL of the lipids were then transferred into a black flat-bottom 96-well plate to generate lipid layers by solvent evaporation at 20 ^oC overnight in a sterile fume hood. **Apotracker Red** was reconstituted at 1 μM in sterile PBS, added to the wells and incubated at 25 ^oC for 40 min. The limit of detection (LoD) of **Apotracker Red** for PtdSer was determined by fluorescence titration of serial dilutions of PtdSer layers after incubation with **Apotracker Red** (1 μM, PBS) at 25 ^oC for 40 min. The LoD was calculated using the equation LoD = $(3 \times \sigma)/k$, where σ is the standard deviation of blank solutions and k is the slope of the linear regression fit. Data was recorded on Cytation 3 (exc: 530 nm).

Isolation of neutrophils from human peripheral blood. *Ex vivo* experiments were performed with neutrophils isolated freshly isolated from the human peripheral blood of healthy donors. Work with human peripheral blood leukocytes complied with all relevant ethical regulations and informed consent was obtained. The study protocol was approved by the Accredited Medical Regional Ethics Committee (AMREC, reference number 20-HV-069) at the University of

Edinburgh. Human peripheral blood neutrophils were isolated as previously described.[3] Briefly, whole blood was drawn into tubes containing anti-coagulant 3.6% sodium citrate (final concentration: 0.4% (w/v)) and centrifuged at 350 g for 20 min at r.t. with lowest acceleration and no brake. Platelet-rich plasma was removed, and leukocytes separated from erythrocytes by 0.6% dextran sedimentation in saline for 30 min at r.t. The upper layer was further fractionated using an isotonic discontinuous Percoll density gradient. Neutrophils were harvested from the 63% and 72.9% interface and cultured in Iscove's Modified Dulbecco's Medium (IMDM, Gibco) in 5% human AB serum for 18 h at 37°C, 5% CO_2 to induce spontaneous apoptosis.

Cell culture. MCF-7, A549, LnCap, MDA-MB-231, Jurkat T cells and PC3 cells were obtained from American Type Culture Collection (ATCC). HT29 cells were purchased from Merck (Sigma Aldrich, UK). Mouse Mammary Tumour Virus–Polyoma Middle T antigen (MMTV-PyMT) cells were provided by the lab of Mikala Egeblad (CSHL, US). MMTV-PyMT, MCF-7, LnCap, MDA-MB-231 and Jurkat T cells were cultured in Roswell Park Memorial Institute (RPMI) 1640 Medium supplemented with 10% fetal bovine serum (FBS), 100 U mL^{-1} penicillin and 0.1 mg mL^{-1} streptomycin. A549 and PC3 cells were cultured in Dublecco's modified Eagle's medium (DMEM) supplemented with 10% fetal bovine serum (FBS), 100 U mL^{-1} penicillin and 0.1 mg mL^{-1} streptomycin.

Flow cytometry drug screen. To evaluate the proliferation of cancer cells, labelling with CellTrace™ Violet was performed according to the manufacturer's instructions. Briefly, cells were resuspended at 6×10^6 cells mL^{-1} and incubated for 30 min with 1 µM CellTrace™ Violet. After centrifugation (300 g, 5 min, r.t.), cell lines were plated on sterile 96-well flat-bottom plates. On the following day, drugs were dissolved in DMF at 10 mM, diluted to the indicated working concentrations and incubated with the cells for 24 or 48 h, as indicated. On the final day, cells

were centrifuged (300 g, 5 min, r.t.), washed once with PBS before addition of 0.05% Trypsin, 0.02% ethylenediaminetetraacetic acid (EDTA) in Hank's balanced salt solution (HBSS) and incubation at 37°C for 5 min. Detached cells were combined in 96-well round bottom plate followed by **Apotracker Red** staining. Histograms and mean fluorescence intensities of apoptotic cells were measured on a 5L LSR flow cytometer under the following excitation/emission filters: **Apotracker Red** (561/610 nm), Annexin V-AF647 (647/670 nm). Data were acquired using the FACS Diva software and analysed using the FlowJo X software.

Induction of apoptosis in Jurkat T cells. Jurkat T cells were induced to undergo apoptosis using 1 µM staurosporine. For confocal imaging, cells were resuspended in RPMI supplemented with 10% FBS, 100 U mL^{-1} penicillin and 0.1 mg mL^{-1} streptomycin and $2.5{\times}10^5$ per well. Cells were imaged between 1 and 3 h after induction of apoptosis and flow cytometry experiments were performed after 2 h of treatment.

Live-cell fluorescence microscopy. Live-cell imaging was performed either on a TCS SP8 confocal microscope (Leica) or in a spinning-disk confocal microscope (Andor). For floating cells, $2.5{\times}10^5$ cells per well were plated in Nunc™ Lab-Tek™ II 8-well chamber slides and allowed to settle for 30 min prior to imaging. For adherent cells, cells were plated at $5{\times}10^4$ cells per well in Nunc™ Lab-Tek™ II 8-well chamber slides on the day prior to imaging. Nuclear staining was performed for 20 min at r.t. with 7 µM Hoechst 33342 prior to imaging together with 150 nM **Apotracker Red** and/or 5 nM Annexin V-AF647. Hoechst 33342 (exc: 405 nm), **Apotracker Red** (exc: 561 nm), Annexin V-AF647 (exc: 633 nm).

MMTV-PyMT breast cancer mouse model and intravital imaging of cisplatin-induced apoptosis. The study protocol was approved by the Institutional Animal Care and Use Committee (IACUC) at Cold Spring Harbor and performed at the Cold Spring Harbor Laboratory

Animal Shared Resources. Animal testing and research complied with the NIH Guide for Care and Use of Laboratory Animals. Mice were housed in a specific-pathogen-free facility with standard husbandry, temperature 19-22 °C, humidity 45-55%. Dark and light cycles were 12 h. Mice were MMTV-PyMT;ACTB-ECFP or MMTV-PyMT;ACTB-ECFP;cfms-EGFP. Tumors were grown to a size between 0.5 and 1 cm in diameter, and then mice were treated with 10 μg g^{-1} cisplatin. Prior to administration of **Apotracker Red**, mice were anaesthetized with 4% isofluorane and kept anesthetized under 1-2 % isofluorane and 21% oxygen balanced with nitrogen at 1 L min^{-1}. Mice were given **Apotracker Red** (5 μM, 100 μL) intravenously and tumour tissue imaging was performed after 1 h or tissue harvested after 2 h. Mice were positioned on the surgical platform with ventral surface facing up and limbs were secured with laboratory tape. Once the animal was secured, hair was removed using an electronic shaver and chemical hair removal. After disinfection of the ventral surface of the mouse with 70% isopropanol wipes and betadine, mammary gland tumours were exposed by subcutaneous ventral midline incision from about 3 mm above the urethra to the xiphoid process. Skin with the inguinal mammary grand was detached gently from the peritoneal cavity. A microscope slide was positioned against the skin flap and the slide was attached to the external surface of the skin using Krazy Glue. The mouse was positioned on imaging stage in the center of a cover glass-covered imaging point. Anesthesia was maintained through a nose cone and a winged intraperitoneal infusion set attached for hourly injection of 50 μL of saline to maintain fluidics. Animal’s vital signs were monitored using an oximeter probe (MouseOx system, Starr Life Sciences). Time-lapse imaging on the spinning disk (Solamere Technologies, Salt Lake City, UT) was acquired with the following settings: ECFP (exc/em: 405/450 nm), EGFP (exc/em: 488/510 nm) and **Apotracker Red** (exc: 561/610 nm). The settings on the two-photon microscope (Nikon) were as followed: ECFP (exc/em: 890/480 nm), SHG (exc/em: 890/420 nm) and **Apotracker Red** (exc/em: 1070/620 nm). After excision of tumours, fixation was carried out for 2 h in 4% PFA at 4°C. Tumours were washed thrice for 10 min with PBS followed by gradual increase with sucrose

solution from 12% sucrose to 30% sucrose every 2 h with a 6% higher sucrose solution. Disposable OCT chambers were used for embedding tumours in OCT on dry ice. OCT solutions were allowed to freeze prior to cutting into 6 μm slides by the Histology Facility of Cold Spring Harbor Laboratories.

NMR Spectra

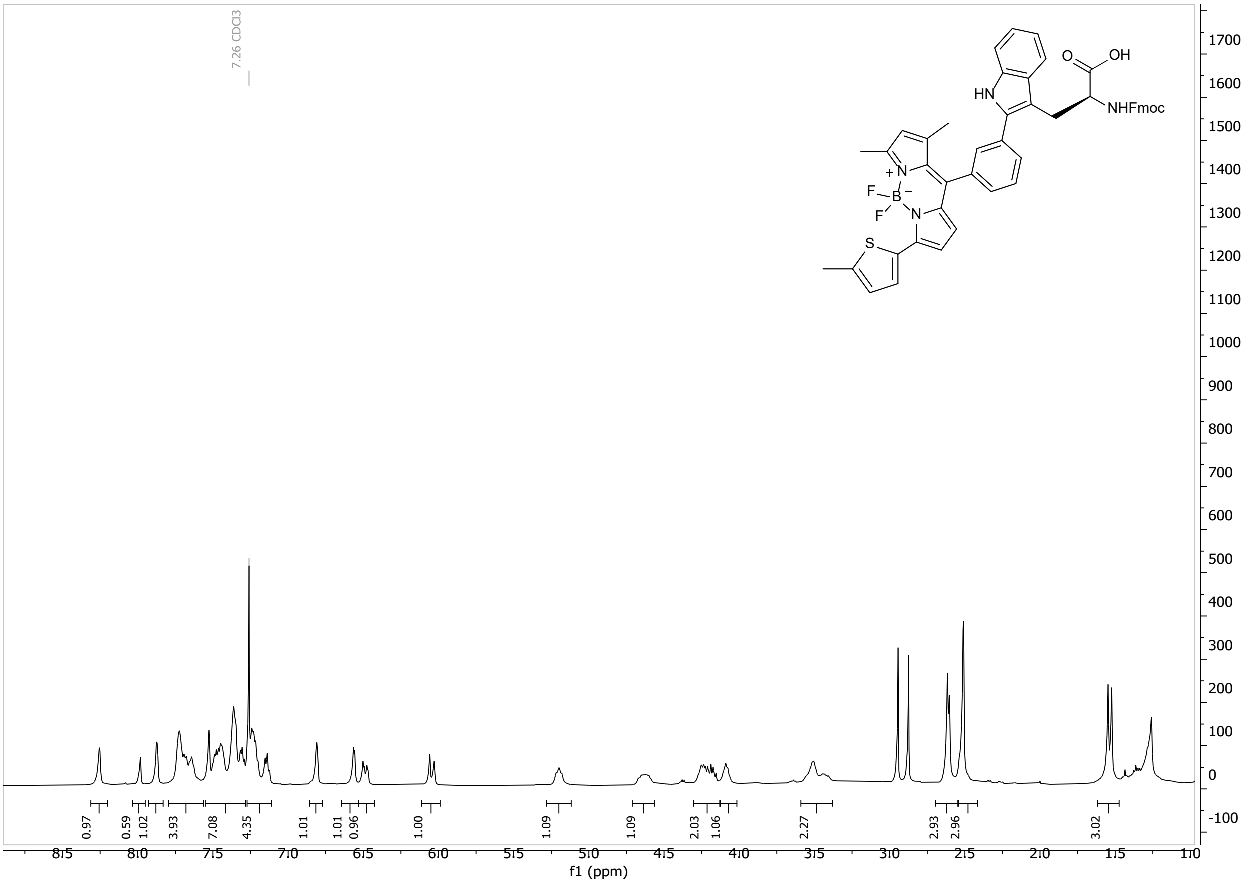
7.26 CDCl3
0.97
0.59
1.02
3.93
7.08
4.35
1.01
1.01
0.96
1.00
1.09
1.09
2.03
1.06
2.27
2.93
2.96
3.02
f1 (ppm)
HN
O
OH
NHFmoc
N
B
F
F
N
S

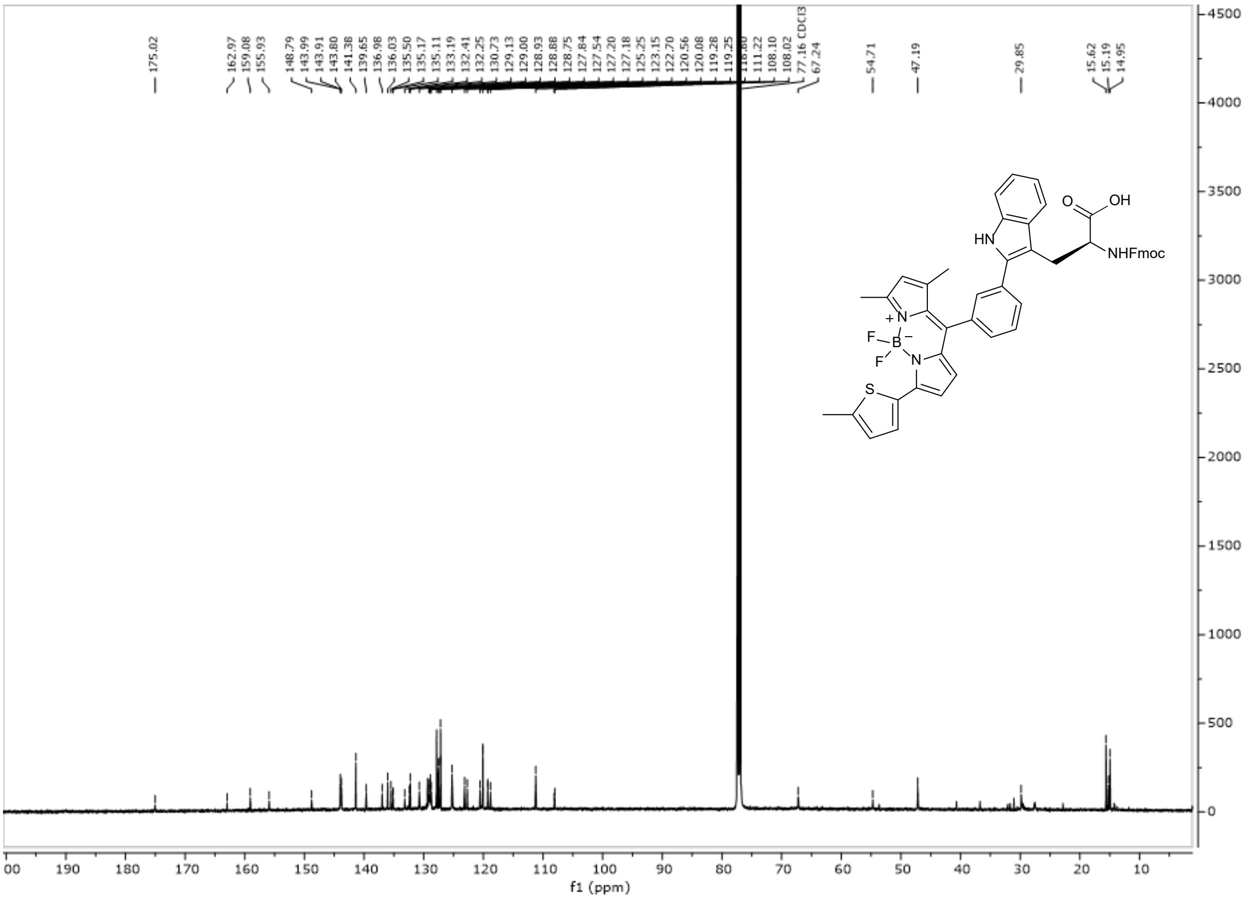
175.02
162.97
159.08
155.93
148.79
143.99
143.91
143.80
141.38
139.65
136.98
136.03
135.50
135.17
135.11
133.19
132.41
132.25
130.73
129.13
129.00
128.93
128.88
128.75
127.84
127.54
127.20
127.18
125.25
123.15
122.70
120.56
120.08
119.28
119.25
111.22
108.10
108.02
77.16 CDCl3
67.24
54.71
47.19
29.85
15.62
15.19
14.95
f1 (ppm)
HN
O
OH
NHFmoc
N
B
F
F
N
S

Supplementary Movie Legends

Movie Legend 1. Two-photon time-course microscopy (up to 3 h) of mammary gland tumours in untreated MMTV-PyMT;ACTB-ECFP mice after injection of **Apotracker Red** (5 μM, red). The co-localisation between ECFP$^+$ cancer cells (green) and **Apotracker Red** appear as yellow signals. SHG signals are presented in blue. Movie played at 10 frames per second. Scale bar: 50 μm.

Movie Legend 2. Two-photon time-course microscopy (up to 3 h) of mammary gland tumour in cisplatin-treated MMTV-PyMT;ACTB-ECFP mice after injection of **Apotracker Red** (5 μM, red). The co-localisation between ECFP$^+$ cancer cells (green) and **Apotracker Red** appear as yellow signals. SHG signals are presented in blue. Movie played at 10 frames per second. Scale bar: 50 μm.

Movie Legend 3. Two-photon time-course microscopy of mammary gland tumour in cisplatin-treated MMTV-PyMT;ACTB-ECFP;cfms-EGFP mice expressing cancer cells (blue), EGFP$^+$ macrophages (green) and after injection **Apotracker Red** (5 μM, red). Movie played at 10 frames per second.

<u>**Supplementary References**</u>

[1] R. Subiros-Funosas, V. C. L. Ho, N. D. Barth, L. Mendive-Tapia, M. Pappalardo, X. Barril, R. Ma, C. B. Zhang, B. Z. Qian, M. Sintes, O. Ghashghaei, R. Lavilla, M. Vendrell, *Chem. Sci.* **2020**, *11*, 1368-1374

[2] J. Arden, G. Deltau, V. Huth, U. Kringel, D. Peros, K. H. Drexhage. *J. Lumin.* **1991**, *48*, 352-358.

[3] C. Haslett, L. A. Guthrie, M. M. Kopaniak, R. B. Johnston, P. M. Henson. *Am. J. Pathol*. **1985**, *119*, 101-110.
